# Tubular Omega‐3 Fatty Acid Receptor FFAR4 Deficiency Aggravated Renal Aging and Chronic Kidney Disease

**DOI:** 10.1111/acel.70546

**Published:** 2026-05-23

**Authors:** Letian Yang, Lei Tang, Jian Li, Dekai Liu, Chunchun Hu, Fan Guo, Lin Lin, Rongshuang Huang, Ping Fu, Liang Ma

**Affiliations:** ^1^ Department of Nephrology, Institute of Kidney Diseases West China Hospital of Sichuan University Chengdu China; ^2^ Department of West Outpatient State Key Laboratory of Oral Diseases & National Center for Stomatology & National Clinical Research Center for Oral Diseases & West China Hospital of Stomatology, Sichuan University Chengdu China

**Keywords:** cellular senescence, chronic kidney disease, free fatty acid receptor 4, kidney aging, omega‐3 polyunsaturated fatty acids, renal fibrosis

## Abstract

Aging leads to renal function decline and increases the risk of chronic kidney disease (CKD). Omega‐3 polyunsaturated fatty acids (PUFAs) are essential fatty acids for humans, exerting their functions via free fatty acid receptor 4 (FFAR4). Clinical studies indicate that omega‐3 PUFAs supplementation shows benefits for the elderly population and CKD patients, but these results remain controversial. Herein, we found that omega‐3 PUFAs alleviated renal fibrosis and tubular senescence in aged mice, adenine diet‐induced CKD mice, and unilateral ureteral obstruction (UUO) mice. Meanwhile, omega‐3 fatty acid receptor FFAR4 expression in tubular epithelial cells (TECs) were down‐regulated in the old population and CKD patients, positively correlated with renal dysfunction. Systemic or TEC‐specific knockout of FFAR4 aggravated renal aging and CKD in mice. Mechanically, FFAR4 agonism increases the production of endogenous PPARγ activator 15‐deoxy‐∆12,14‐Prostaglandin J2 (15d‐PGJ2), and improves PPARγ‐dependent tubular epithelial cell senescence, which was indicated by anti‐aging marker Klotho expression, senescence‐associated β‐galactosidase (SA‐β‐gal) activity, and profibrotic factor TGF‐β1 secretion. The study demonstrated a novel role of FFAR4 from senescent TECs on fibroblast activation via paracrine effects and highlighted the therapeutic effects of omega‐3 PUFAs with their receptor FFAR4 as an attractive drug target against renal aging and CKD.

## Graphical abstract

This study illustrates that omega‐3 PUFAs with their receptor FFAR4 alleviate tubular senescence and fibrosis in aged and fibrotic kidneys. Furthermore, we indicate that tubular FFAR4 improves renal senescence via 15d‐PGJ2‐PPARγ‐Klotho signaling, and suppresses kidney fibrosis by senescent tubular cell‐driven fibroblast activation. Here, we propose a therapeutic strategy of omega‐3 PUFAs, but also highlight that tubular FFAR4 is a potential target against renal aging and CKD.

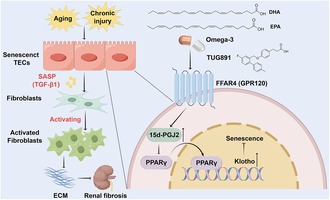

## 1 Introduction

With aging of global population, the proportion of older adults is increasing dramatically (Guo et al. 2022). Kidney is one of the organs most easily affected by aging. Aged kidneys undergo obvious structural and functional changes (Schmitt and Melk 2017). In addition, advanced age is regarded as a common risk factor for chronic kidney disease (CKD) (GBD Chronic Kidney Disease Collaboration 2020; KDIGO 2024). At present, therapeutic drugs are lacking for the treatment of renal aging and CKD, leading to the immense health and economic burden (Chen et al. 2024).

Renal fibrosis is recognized as a common pathologic feature of renal aging and CKD progression, with complex pathogenesis (Schmitt and Melk 2017; Ha et al. 2024; Wu, Lin, et al. 2024; Lai et al. 2024). Previous studies mainly focused on epithelial‐to‐mesenchymal transition (EMT), which is recently challenged. The current view holds that abnormal fibroblast activation is the major event of renal fibrosis (Huang et al. 2023). Various secreted soluble factors are believed to induce fibroblast activation, including transforming growth factor‐β1 (TGF‐β1), fibroblast growth factors 2 (FGF2), etc.

Cellular senescence is defined as an irreversible cell cycle arrest process, grouped into replicative senescence (physiological aging) and premature senescence (senescence in pathological conditions). Premature senescence is induced by several stressors, such as DNA damage, oxidative stress, etc. (Di Micco et al. 2021; Suryadevara et al. 2024; Zhang et al. 2022). Cellular senescence of renal parenchymal cells, such as tubular epithelial cells (TECs), glomerular endothelial cells, and podocytes, has been observed in aged kidneys (Suryadevara et al. 2024). Notably, TECs are widely thought to be most vulnerable to undergoing cellular senescence (Ha et al. 2024; Suryadevara et al. 2024; Docherty et al. 2019; Huang et al. 2022; Li et al. 2023; Yamamoto and Isaka 2024). Senescent cells produce numerous cytokines, collectively called the senescence‐associated secretory phenotype (SASP), such as TGF‐β1, contributing to multi‐organ fibrosis (O'Reilly et al. 2024). It is currently accepted that injured TECs would release multiple pro‐fibrotic factors to activate fibroblasts. This crosstalk between TECs and fibroblasts is considered an important driver of renal fibrosis in CKD (Li et al. 2022, 2023; He et al. 2024; Livingston et al. 2023). However, the underlying mechanisms of senescence‐related renal fibrosis are not well understood and effective interventions are still lacking.

Omega‐3 polyunsaturated fatty acids (PUFAs) mainly include eicosapentaenoic acid (EPA; 20:5 ω‐3) and docosahexaenoic acid (DHA; 22:6 ω‐3), participating in multiple biological processes. Recently, the anti‐senescent effects of omega‐3 PUFAs have attracted great attention. Omega‐3 PUFAs treatment effectively mitigates telomere shortening and slows biological aging across epigenetic clocks in older adults (Bischoff‐Ferrari et al. 2025; Farzaneh‐Far et al. 2010; Ogłuszka et al. 2022; O'Callaghan et al. 2014; Wu, Jia, et al. 2024). Of note, several clinical studies and animal experiments indicate that omega‐3 PUFAs contribute to the improvement of age‐related cognitive impairment, sarcopenia, and cardiovascular diseases (Gaengler et al. 2024; Cornish et al. 2022; Zhang, Yuan, et al. 2024; Pan et al. 2024; Xiong et al. 2024; Campanari et al. 2024). However, research on the role of omega‐3 PUFAs in modulating renal aging remains limited. Moreover, the therapeutic potential of omega‐3 PUFAs in CKD has been extensively investigated; however, its efficacy remains controversial due to conflicting evidence from clinical trials and unresolved mechanistic insights (de Boer et al. 2019; Ong et al. 2023). Collectively, their results underscore the imperative to explicate the therapeutic effects and underlying mechanisms of omega‐3 PUFAs in renal aging and CKD (Figure S1).

Omega‐3 PUFAs exert their biological functions via free fatty acid receptor 4 (FFAR4), a member of the G protein‐coupled receptor (GPCR) family (also named GPR120). The roles of FFAR4 have been demonstrated in a variety of diseases, such as diabetes, inflammatory bowel disease, and hepatic failure (Carullo et al. 2021; Yang, Liu, et al. 2022; Yu et al. 2021; Yang, Wang, et al. 2022; Wei et al. 2021; Yin et al. 2025; Huang et al. 2020). However, the roles of tubular FFAR4 in senescence‐related renal fibrosis have not been well elaborated, and the underlying mechanisms are still unclear. Investigating the role of FFAR4 in aging and CKD may elucidate the molecular mechanisms underlying the biological effects of omega‐3 PUFAs, thereby guiding the clinical application. Furthermore, discrepancies in clinical trials have partially restricted the use of omega‐3 PUFAs. Hence, the development of drugs targeting omega‐3 receptor FFAR4 represents a highly promising direction to intervene in renal aging and CKD.

In this study, we investigated that omega‐3 PUFAs and their receptor FFAR4 exhibit reno‐protective, anti‐fibrotic, and anti‐senescent effects in aged mice and diseased mice. These results highlight that omega‐3 PUFAs alleviate age‐related renal function decline and protect against CKD, suggesting that FFAR4 may represent an attractive target against renal aging and CKD.

## 2 Methods

### 2.1 Human Kidney Biopsy Samples

Human kidney sections of CKD patients were obtained from diagnostic renal biopsies performed at West China Hospital of Sichuan University. The kidney tissues of elderly and control subjects were obtained from patients without kidney diseases, undergoing tumor nephrectomies at West China Hospital of Sichuan University. The protocol was approved by the Biomedical Ethics Committee of West China Hospital of Sichuan University. The clinical information of enrolled patients was summarized in Tables S1 and S2.

### 2.2 Chemicals and Antibodies

Omega‐3 PUFAs capsules were purchased from Sichuan Gowell Pharmaceutical Co. Ltd. (Chengdu, China), containing 40 g EPA and 32 g DHA per 100 g. TUG891 (SML1914) was purchased from Sigma‐Aldrich (St. Louis, MO, USA). Adenine (A8626, purity ≥ 99%) was purchased from Sigma‐Aldrich (St. Louis, MO, USA). Human recombinant TGF‐β1 (100‐21C 50UG) was purchased from Thermo Fisher Scientific (Waltham, MA, USA). T0070907 (HY‐13202) was purchased from MCE (Shanghai, China). The primary antibodies used in this study are shown in Table S3. Horseradish peroxidase‐conjugated secondary antibodies and LTL (FL‐1321‐2) were purchased from Thermo Fisher Scientific (Waltham, MA, USA) and Vector Laboratories (Newark, California, USA), respectively.

### 2.3 Animal Experiments

The ethical approval of animal experiments was obtained from the Experimental Animal Ethics Committee of West China Hospital of Sichuan University (approval number: 2020192A). Wild type C57BL/6J mice, systemic FFAR4 deletion mice (FFAR4‐KO) and TEC‐specific FFAR4 deletion mice (FFAR4^TecKO^) were purchased from Gempharmatech Co. Ltd. (Nanjing, China) (Figures S14–S17). All mice were grouped randomly (6 mice per group). For the natural aging model, male mice aged 3 and 22 months old were used. Omega‐3 PUFAs were added to regular chew diet at a ratio of 0.0242 g:1 g, corresponding to the dose of 2 g omega‐3 PUFAs/kg/d. For the CKD model, adenine diet‐induced CKD mice were fed with the regular chew diet with 0.2% adenine (Research Diets Inc., Brunswick, NJ) for 2 weeks, corresponding to the dose of 160 mg adenine/kg/day. Unilateral ureteral obstruction (UUO) mice model was generated as we previously described (Zhang, Su, et al. 2024). Omega‐3 PUFAs was administered at a dose of 2 g/kg daily by gavage.

### 2.4 Renal Function and Histopathologic Evaluation

Blood samples of mice were collected and centrifuged to obtain serum at room temperature (3000 rpm, 20 min). An automatic biochemical analyzer (Mindray BS‐240, Shenzhen, China) was used to measure serum creatine (Scr) and blood urea nitrogen (BUN) levels. Renal cortex tissues were flash‐frozen in liquid nitrogen, then stored at −80°C. For the middle part of the kidney, a portion was fixed in 10% formaldehyde (50‐00‐0, Chron Chemicals, Chengdu, China), dehydrated, embedded in paraffin, and cut into sections with a thickness of 4 μm for hematoxylin–eosin (H&E) staining and Masson staining. Others were embedded in the optimal cutting temperature (OCT) compound, flash‐frozen in liquid nitrogen, then stored at −80°C. Images of kidney sections were taken and analyzed using a Nikon Eclipse Ni‐E microscope (Nikon, Tokyo, Japan), equipped with a CMOS camera ORCA‐Flash4.0 and NIS‐Elements Version D 3.10 software. Tubular injury scores were determined on a semiquantitative grade scale: 0, normal; 1, < 25% damage; 2, 25%–50% damage; 3, 51%–75% damage; and 4, > 75% damage. The positive area of Masson staining was assessed by collagen volume fraction (CVF) via ImageJ software (version 1.51, Wayne Rasband, NIH, USA).

### 2.5 Transdermal Glomerular Filtration Rate Measurement

The transdermal GFR (TGFR) system is designed to assess kidney function by measuring the clearance rate of a fluorescent tracer agent. It consists of three key components: the TGFR sensor, the TGFR monitor, and FITC‐Sinistrin (MediBeacon, Creve Coeur, MO, USA). The system operates by transdermally recording the fluorescence intensity of FITC‐Sinistrin over time via a skin‐placed sensor. The TGFR sensor captures data at a rate of 2.5 readings per second, and the TGFR monitor subsequently computes and displays the average session TGFR reading. The mice were shaved one day in advance. On the day of measurement, the TGFR sensor was placed on the mouse's skin, then FITC‐Sinistrin was injected via the tail vein (7 mg/100 g body weight). During the measurements, the mice were allowed to move freely to ensure a natural behavioral state. 120 min later, the recording was read and analyzed using MediBeacon Studio V2 software (MediBeacon, Creve Coeur, MO, USA). The TGFR was calculated by analyzing the concentration time curves of FITC‐Sinistrin.

### 2.6 Cell Culture and Treatments

HK‐2 cells were purchased from ATCC agency Shanghai Limai Biological Engineering Co. Ltd. (Shanghai, China) and cultured in minimum essential medium (MEM) (ThermoFisher Scientific) with 10% fetal bovine serum (FBS) (Hyclone, Beijing, China). For the TGF‐β1‐induced senescence study, HK‐2 cells were incubated in MEM medium with 0.5% FBS, supplemented with 10 ng/mL TGF‐β1 for 48 h. To assess the therapeutic effects of TUG891 treatment, cells were incubated with 10 μM TUG891 and 10 ng/mL TGF‐β1 simultaneously for 48 h. For the hydrogen peroxide (H_2_O_2_)‐induced senescence study, HK‐2 cells were treated with 400 μmol/L H_2_O_2_ for 2 h, then the medium was replaced with fresh MEM containing 10% FBS, and cells were cultured for an additional 72 h. TUG891 (10 μM) was administered throughout the entire culture period. To explore the effects of peroxisome proliferator‐activated receptor γ (PPARγ) inhibition, cells were incubated with T0070907 (a PPARγ antagonist), 10 μM TUG891, and 10 ng/mL TGF‐β1 simultaneously for 48 h. The protocol of siRNA transfection was described previously (Yang, Wang, et al. 2022). The sequences of FFAR4 siRNA and NC siRNA were listed in the Supplementary Methods.

### 2.7 The Supernatant From HK‐2 Cells Collection and Treatment of NRK‐49F Fibroblasts

HK‐2 cells were subjected to 10 ng/mL TGF‐β1 treatment for 48 h. Then the culture media for TGF‐β1‐treated cells were replaced with fresh media free of TGF‐β1 and incubated for 24 h to collect supernatant. NRK‐49F fibroblasts were purchased from ATCC agency Shanghai Limai Biological Engineering Co. Ltd. and cultured in dulbecco's modified eagle medium (DMEM) (Thermo Fisher Scientific) with 10% FBS. NRK‐49F fibroblasts were incubated with supernatant from HK‐2 cells (1 mL supernatant diluted in 9 mL DMEM) for 48 h.

### 2.8 The Measurements of TGF‐β1 and 15‐Deoxy‐∆12,14‐Prostaglandin J2

The content of TGF‐β1 and 15‐deoxy‐∆12,14‐Prostaglandin J2 (15d‐PGJ2) in supernatant from HK‐2 cells was measured with enzyme linked immunosorbent assay (ELISA) kits (PT880, Beyotime Biotechnology, Shanghai, China; ADI‐900‐023, Enzo Life Sciences, Farmingdale, NY, USA) according to the manufacturer's guidelines. Renal 15d‐PGJ2 levels were quantified by high‐performance liquid chromatography coupled with tandem mass spectrometry (HPLC‐MS/MS), with methodological details provided in Supplementary Methods.

### 2.9 Immunohistochemistry

Paraffin‐embedded renal tissues were cut into sections with a thickness of 4 μm, then underwent deparaffinization, rehydration, and antigen retrieval. The kidney sections were then blocked with 2.5% goat serum for 1 h at room temperature, then incubated with primary antibodies diluted 1:200 in goat serum at 4°C overnight. The sections were washed three times in phosphate‐buffered saline (PBS) and stained using the VECTASTAIN ABC Kit (Vector, Burlingame, CA, USA). Images of kidney sections were taken and analyzed using a Nikon Eclipse Ni‐E microscope (Nikon, Tokyo, Japan), equipped with a CMOS camera ORCA‐Flash4.0 and NIS‐Elements Version D 3.10 software. Quantitative analysis in immunohistology was performed with Image‐J software.

### 2.10 Immunofluorescence

Paraffin‐embedded renal tissues were cut into sections with a thickness of 4 μm, then underwent deparaffinization, rehydration, and antigen retrieval. The kidney sections were then blocked with 10% horse serum for 1 h at room temperature, then incubated with primary antibodies diluted 1:100 in 10% horse serum at 4°C overnight.

Horseradish peroxidase‐conjugated secondary antibodies diluted 1:200 were used for 1 h. LTL diluted 1:200 was used to label proximal renal tubules. The kidney sections were washed three times in PBS, then stained with DAPI diluted 1:200 (D8200, Solarbio, Beijing, China). Images of kidney sections were taken and analyzed using a Nikon Eclipse Ni‐E microscope (Nikon, Tokyo, Japan), equipped with a CMOS camera ORCA‐Flash4.0 and NIS‐Elements Version D 3.10 software.

For cell immunofluorescent staining, the cultured cells were washed with PBS three times and fixed with 4% polyformaldehyde for 15 min at room temperature. Next, the cells were permeabilized with 0.5% Triton X‐100 (Sigma‐Aldrich) for 30 min and blocked with 5% bovine serum albumin (BSA) for 1 h at room temperature. The subsequent steps were the same as above. Images were taken and analyzed using a Nikon Ti‐Eclipse microscope (Nikon, Tokyo, Japan), equipped with a CMOS camera ORCA‐Flash4.0 and NIS‐Elements Version D 3.10 software.

### 2.11 SA‐β‐Gal Staining

The SA‐β‐gal staining of kidney sections and cultured cells was performed using a kit (C0602, Beyotime Biotechnology, Shanghai, China) according to the manufacturer's guidelines. Images were taken and analyzed using a Nikon Ti‐Eclipse microscope and a Nikon Eclipse Ni‐E microscope (Nikon, Tokyo, Japan). Subsequent quantitative analysis was performed with Image‐J software.

### 2.12 Lipofuscin Staining

Lipofuscin staining of kidney sections was performed using a commercial kit (S0209, BIOSS Biotechnology, Beijing, China) following the manufacturer's instructions. Images of kidney sections were taken and analyzed using a Nikon Eclipse Ni‐E microscope (Nikon, Tokyo, Japan), equipped with a CMOS camera ORCA‐Flash4.0 and NIS‐Elements Version D 3.10 software.

### 2.13 RNA‐Sequencing

RNA‐sequencing was performed as previously described (Yang, Wang, et al. 2022). The short summary is as follows. Frozen kidneys (Control group: *n*=3 per group. Model group: *n*=4 per group) were randomly selected for RNA‐sequencing. Total RNA of kidneys was extracted with TRIzol reagent (Invitrogen, Carlsbad, CA, USA), followed by sample integrity, quality, and purity examination. Construction of libraries and sequencing were performed in LC‐BIO Bio‐Tech Ltd. (Hangzhou, China).

### 2.14 Western Blot

Western blot analysis was performed as previously described (Wei et al. 2021). Immunoblots were visualized with a ChemiDoc‐MP imaging system (Clinx Science, Shanghai, China) and quantified using ImageJ software (version 1.51, Wayne Rasband, NIH, USA).

### 2.15 Quantitative Real‐Time PCR Analysis

The protocol of total RNA isolation, reverse transcription and quantitative real‐time PCR (RT‐qPCR) was described previously (Yang, Wang, et al. 2022). The primers used for the target genes are listed in Table S4. Normalization of gene expression was done using glyceraldehyde‐3‐phosphate dehydrogenase (GAPDH) expression as a reference and calculated by CFX Manager Software (Bio‐Rad, Hercules, CA, USA).

### 2.16 Statistical Analysis

Data are displayed as the mean ± SD. The differences between two groups were examined by two‐tailed Student's *t*‐test (for parametric data) or Mann–Whitney *U* test (for non‐parametric data), and differences between more than two groups were examined with one‐way ANOVA (for one experimental parameter) or two‐way ANOVA (for two experimental parameters), with subsequent analysis by Tukey's multiple comparisons test. GraphPad Prism 9.0 (GraphPad Software, San Diego, CA, USA) was utilized for performing all statistical analyses.

## 3 Results

### 3.1 Omega‐3 PUFAs Protected Aging‐Related Structural and Functional Deterioration in Aged Kidneys

**FIGURE 1:**
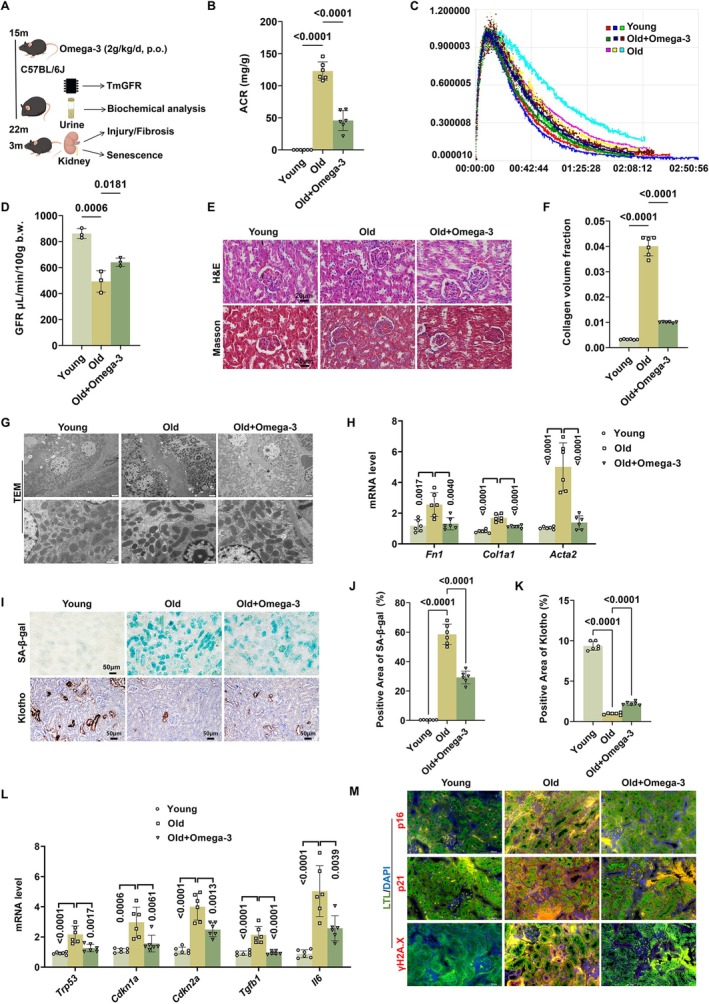
Omega‐3 PUFAs attenuated aging‐related renal function decline, tubulointerstitial fibrosis and tubular senescence in aged mice. (A) Schematic representation of animal experiment. 15‐month‐old wild‐type mice were treated with omega‐3 PUFAs (2 g/kg/day, p.o.) for 7 months (*n* = 6 per group). Transdermal glomerular filtration rate, urine albumin creatinine ratio, tubulointerstitial fibrosis and tubular senescence were assessed at end‐point. (B) Urine albumin creatinine ratio of mice in each group (*n* = 6 per group). (C) Concentration time curves of mice in each group (*n* = 3 per group), after i.v. injection of 7 mg/100 g FITC‐Sinistrin into mice measured transcutaneously. The slope of the curves during the excretion period demonstrates the capacity of renal clearance of FITC‐Sinistrin and suggests appropriateness of transcutaneous measurement for GFR determination (*n* = 3 per group). (D) Transdermal glomerular filtration rate of mice in each group calculated from the concentration time curves using MediBeacon Studio V2 software (*n* = 3 per group). (E) Representative images of H&E staining and Masson staining of kidney sections in each group. (F) Collagen volume fraction of kidneys in each group (*n* = 6 per group). (G) Representative images of transmission electron microscope of kidney sections in each group. (H) Relative mRNA levels of fibrosis‐related genes *Fn1*, *Col1a1*, and *Acta2* in kidneys in each group (*n* = 6 per group). (I) Representative images of SA‐β‐gal staining and immunohistochemical staining of Klotho of kidney sections in each group. (J) Quantification of SA‐β‐gal‐positive areas of kidney sections in each group (*n* = 6 per group). (K) Quantification of immunohistochemical staining of Klotho in kidneys of mice in each group (*n* = 6 per group). (L) Relative mRNA levels of senescence‐related genes *Trp53*, *Cdkn1a*, *Cdkn2a*, *Tgfb1*, and *Il6* in kidneys in each group (*n* = 6 per group). (M) Representative images of double immunofluorescence for p16/p21/γH2A.X and LTL of kidneys sections in each group. Markers: p16/p21/γH2A.X, red; LTL: green; DAPI, blue. Overlay images (p16/p21 + LTL) appear yellow and purple, due to the colocation of red/green markers and red/blue markers, respectively. Overlay images (γH2A.X + DAPI) appear purple due to the colocation of red and blue markers. Data are presented as mean ± SD. ACR, albumin creatinine ratio; GFR, glomerular filtration rate.

To further clarify the therapeutic efficacy of omega‐3 PUFAs in aged kidneys, we treated 15‐month‐old mice with omega‐3 PUFAs for 7 months (Figure 1A). As shown in Figure 1B–F, old mice presented with reduced transdermal glomerular filtration rate (TGFR), increased urinary protein excretion, tubular atrophy and tubule dilation, which were relieved after the treatment of omega‐3 PUFAs. Ultrastructural changes of TECs were also evident in aged kidneys (partially disrupted nuclear membranes, chromatin condensation, increase in secondary lysosomes, and swollen mitochondria with unclear cristae), significantly restored in the omega‐3 PUFAs group (Figure 1G). Furthermore, omega‐3 PUFAs treatment also reduced collagen deposition and attenuated the increase of the mRNA levels of profibrotic markers (*Fn1*, *Col1a1*, and *Acta2*) in aged kidneys (Figure 1H).

We then assessed cellular senescence of TECs in aged kidneys. Senescence‐associated β‐galactosidase (SA‐β‐gal) activity has been regarded as the standard for determining senescent cells, and Klotho is a well‐known anti‐aging protein synthesized by renal tubules (Chen et al. 2024). As shown in Figure 1I–K, SA‐β‐gal‐positive tubules were increased and Klotho‐positive tubules were decreased in aged kidneys, with the obvious improvement in omega‐3 PUFAs treatment group. Additionally, the mRNA levels of cellular senescence markers (*Trp53*, *Cdkn1a*, and *Cdkn2a*) and SASP (*Il6* and *Tgfb1*) were significantly decreased following omega‐3 PUFAs treatment (Figure 1L). Afterwards, to further verify the localization of senescent cells, co‐immunofluorescence (IF) staining of cellular senescence markers p16, p21, and phosphorylated histone H2A.X (γH2A.X) with *lotus tetragonolobus* lectin (LTL, a marker of proximal renal tubule) was performed. Compared with other cell types, the degree of cellular senescence was markedly greater and the anti‐senescence effects of omega‐3 PUFAs were more potent in TECs (Figure 1M).

### 3.2 Omega‐3 PUFAs Exerted Reno‐Protective and Anti‐Senescence Effects in CKD Mice

CKD represents a state of premature senescence. Previous studies have investigated the roles of omega‐3 PUFAs in CKD, yet their findings remain controversial. And no study explored the mechanism whereby omega‐3 PUFAs exerts reno‐protective effects in CKD via improving tubular senescence. Hence, we next investigated the effects of omega‐3 PUFAs in adenine diet‐induced CKD and UUO mice (Figures S2A and S3A). As presented in Figure S2B–H, omega‐3 PUFAs restored Scr and BUN levels, attenuated renal tubular injury, and alleviated renal fibrosis of adenine‐induced mice. In addition, adenine‐induced tubular senescence was obviously alleviated following omega‐3 PUFAs treatment (Figure S2I–K). Afterwards, the reno‐protective and anti‐senescence effects of omega‐3 PUFAs were also verified in UUO mice (Figure S3B–G).

### 3.3 Tubular Omega‐3 Fatty Acid Receptor FFAR4 Was Correlated With Aging and Chronic Injury‐Related Renal Function Decline

**FIGURE 2:**
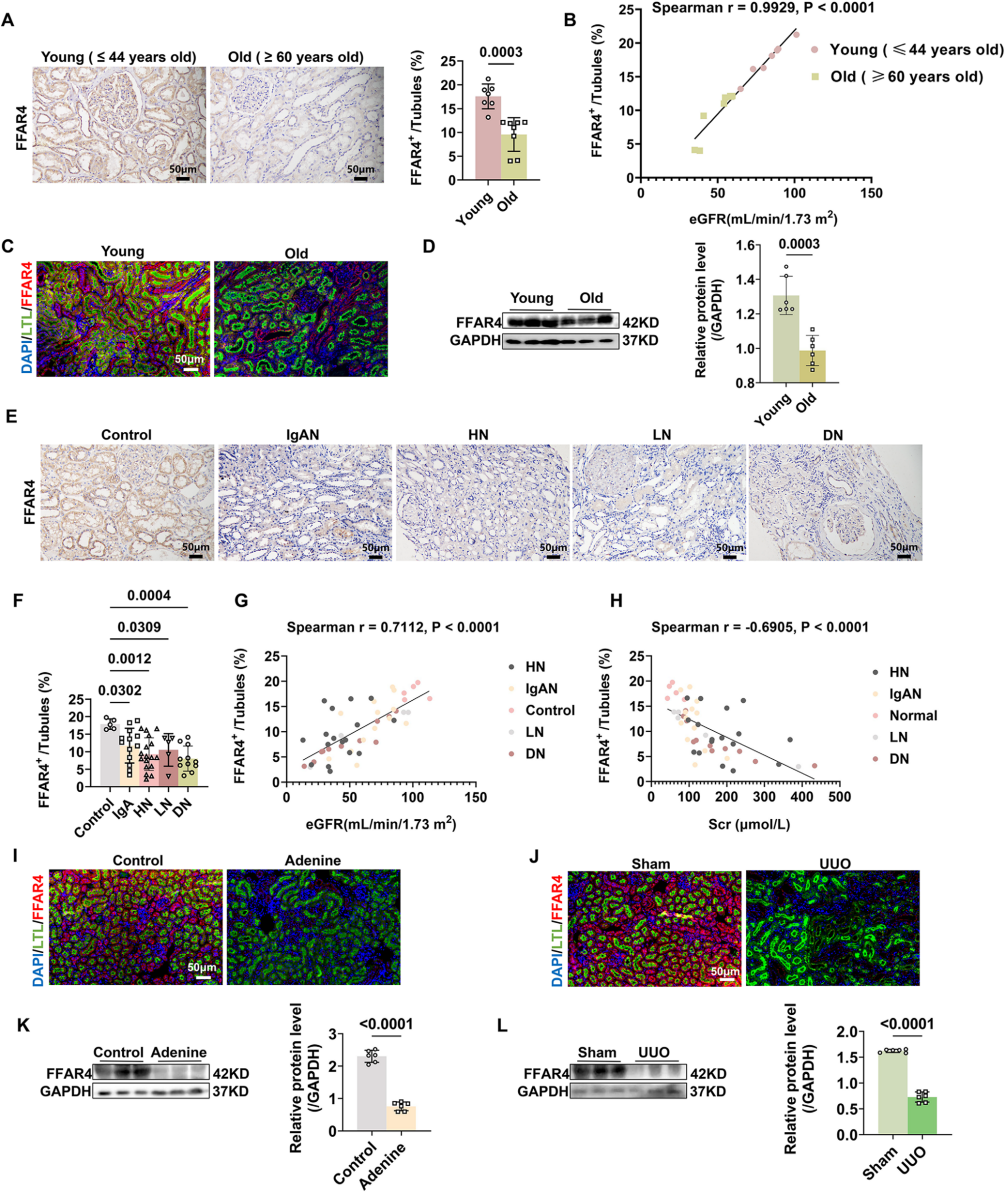
Decreased expression of FFAR4 in TECs was positively correlated with renal function decline in aging and CKD. (A) Representative images (left) and corresponding quantification (right) of immunohistochemical staining of FFAR4 in kidneys of young (age 18–44 years, *n* = 7) and old (age ≥ 60 years, *n* = 8) subjects. (B) Spearman's correlation analysis between protein expressions of tubular FFAR4 and eGFR in young and aged subjects. Each point represents values from a single individual from young or old group. (C) Representative images of double immunofluorescence staining for FFAR4 and LTL in kidneys of 22‐month‐old mice and 3‐month‐old mice mice. Markers: FFAR4, red; LTL: green; DAPI, blue. Overlay images (FFAR4 + LTL) appear yellow due to the colocation of red and green markers. (D) Western Blot analysis of FFAR4 protein expressions in aged kidneys of mice (*n* = 6 per group). (E) Representative images and corresponding semi‐quantification (F) of immunohistochemical staining of FFAR4 in kidneys of healthy controls (*n* = 5) and patients with IgAN (*n* = 13), HN (*n* = 17), LN (*n* = 5), or DN (*n* = 11). (G, H) Spearman's correlation analysis of tubular FFAR4 expressions with eGFR or Scr in healthy controls and CKD patients. Each point represents values from a healthy subject or a CKD patient. (I, J) Representative images of double immunofluorescence staining for FFAR4 and LTL in kidneys of adenine diet‐induced CKD mice and UUO mice. Markers: FFAR4, red; LTL: green; DAPI, blue. Overlay images (FFAR4 + LTL) appear yellow due to the colocation of red and green markers. (K, L) Western Blot analysis of FFAR4 protein expressions in kidneys of adenine diet‐induced CKD mice and UUO mice (*n* = 6 per group). Data are presented as mean ± SD. DN, diabetic nephropathy; eGFR, estimated glomerular filtration rate; HN, hypertension nephropathy; IgAN, IgA nephropathy; LN, lupus nephropathy; Scr, serum creatine; UUO, unilateral ureteral obstruction.

Having established the significant reno‐protective and anti‐senescence effects of omega‐3 PUFAs in both aged kidneys and CKD models, we subsequently directed our investigation toward elucidating the roles of FFAR4. First, we investigated the expression of tubular FFAR4 in healthy older adults and CKD patients. As presented in Figure 2A,B, FFAR4 protein expression in TECs was significantly decreased in aged human kidneys, associated with the age‐related decline of estimated glomerular filtration rate (eGFR). Furthermore, compared with 3‐month‐old mice, co‐IF staining of FFAR4 and LTL illustrated that FFAR4 protein expression in TECs was obviously decreased in kidneys of 22‐month‐old mice (Figure 2C). Similar trends were also shown in western blot analysis (Figure 2D).

Afterwards, the down‐regulated expression of FFAR4 was also confirmed in renal biopsies from CKD patients with IgA nephropathy (IgAN), hypertension nephropathy (HN), lupus nephropathy (LN), or diabetic nephropathy (DN), compared with tumor‐adjacent normal renal tissues (Figure 2E,F). Meanwhile, tubular FFAR4 expression was positively correlated with eGFR and negatively correlated with Scr in CKD patients (Figure 2G,H). Similarly, the expression of tubular FFAR4 was also decreased in fibrotic kidneys of CKD mice (Figure 2I–L).

### 3.4 Tubular Epithelial Cell‐Specific FFAR4 Deletion Exacerbated Aging‐Related Renal Function Decline and Fibrosis in Mice

**FIGURE 3:**
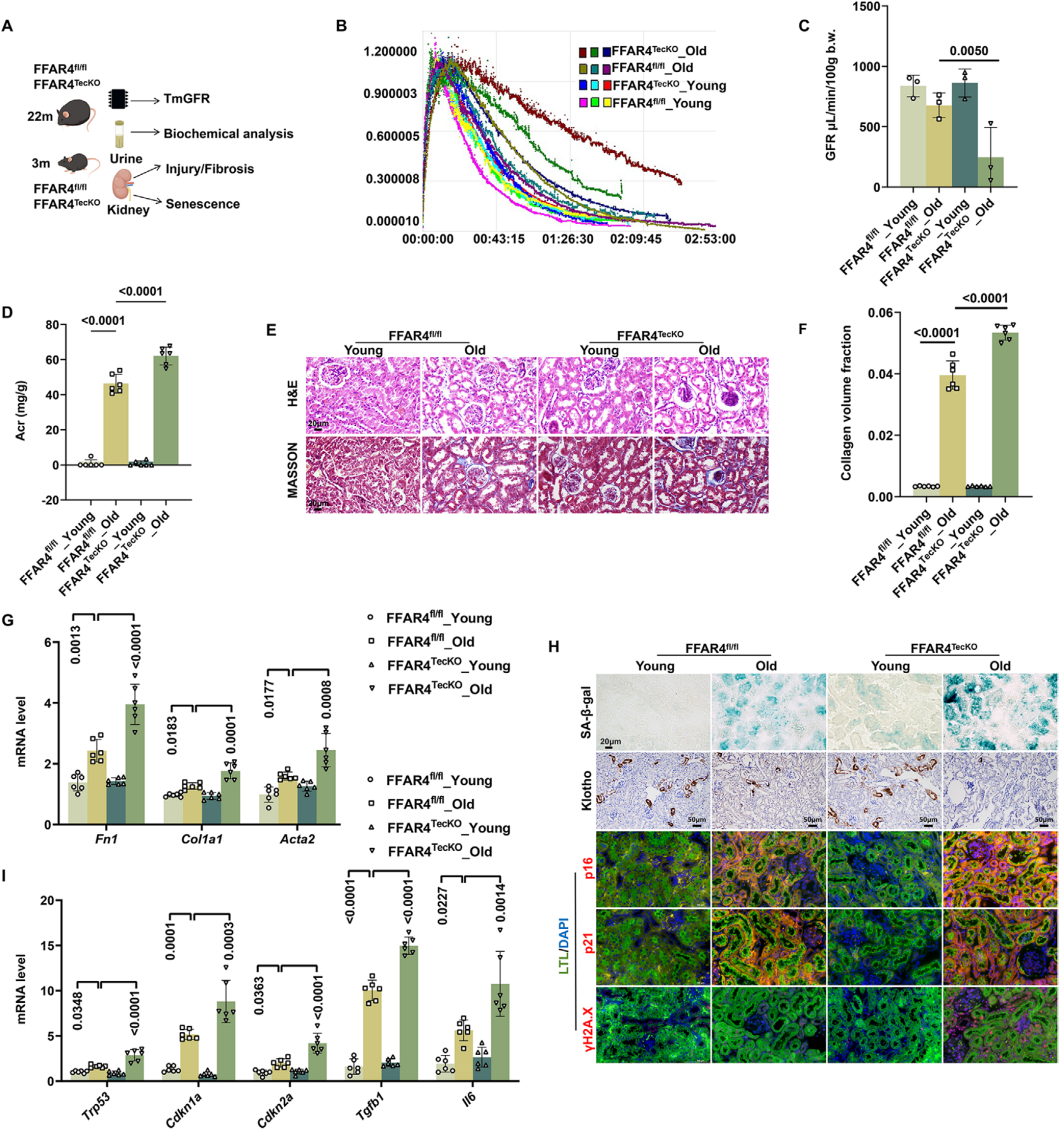
TEC‐specific FFAR4 deletion exacerbated renal function decline and tubulointerstitial fibrosis in aged mice. (A) Schematic representation showing the assessment of renal aging in aged FFAR4^TecKO^ mice and aged FFAR4^fl/fl^ mice (22 months old), compared with young mice (3 months old). (B) Concentration time curves after i.v. injection of 7 mg/100 g FITC‐Sinistrin into mice measured transcutaneously (*n* = 3 per group). (C) Transdermal glomerular filtration rate of mice in each group calculated from the concentration time curves (*n* = 3 per group). (D) Urine albumin creatinine ratio of mice in each group (*n* = 6 per group). (E) Representative images of H&E staining and Masson staining of kidney sections in each group. (F) Collagen volume fraction of kidneys in each group (*n* = 6 per group). (G) Relative mRNA levels of fibrosis‐related genes *Fn1*, *Col1a1*, and *Acta2* of kidneys in each group (*n* = 6 per group). (H) Representative images of SA‐β‐gal staining, immunohistochemical staining of Klotho, and double immunofluorescence for p16/p21/γH2A.X and LTL of kidney sections in each group. Markers: p16/p21/γH2A.X, red; LTL: green; DAPI, blue. Overlay images (p16/p21 + LTL) appear yellow and purple, due to the colocation of red/green markers and red/blue markers, respectively. Overlay images (γH2A.X + DAPI) appear purple due to the colocation of red and blue markers. (I) Relative mRNA levels of senescence‐related genes *Trp53*, *Cdkn1a*, *Cdkn2a*, *Tgfb1*, and *Il6* of kidneys in each group (*n* = 6 per group). Data are presented as mean ± SD. ACR, albumin creatinine ratio; GFR, glomerular filtration rate.

To further specify the effects of tubular FFAR4 in renal aging, FFAR4^TecKO^ mice were utilized to construct an aging model, while FFAR4^fl/fl^ mice were employed as controls (Figure 3A). Compared with old FFAR4^fl/fl^ mice, old FFAR4^TecKO^ mice presented with lower TGFR, higher urinary protein excretion, worse tubular pathological injury, higher mRNA levels of kidney injury markers hepatitis A virus cellular receptor 1 (*Havcr1*) and lipocalin‐2 (*Lcn2*), and exacerbated renal fibrosis (Figure 3B–G and Figure S4). Afterwards, aggravated aging‐induced senescence phenotypes of TECs in FFAR4^TecKO^ mice were also confirmed (Figure 3H,I and Figure S5).

To further characterize the senescence phenotype in FFAR4^TecKO^ mice, we assessed multiple senescence‐associated markers. As shown in Figure 3H,I and Figure S5, the FFAR4^TecKO^ group exhibited significantly exacerbated age‐related tubular senescence compared with the FFAR4^fl/fl^ group, including increased lipofuscin accumulation (a classic hallmark of cellular senescence), elevated SA‐β‐Gal activity, upregulation of p53, p21, p16, IL‐6, and TGF‐β1, and downregulation of Klotho.

### 3.5 Systemic or TEC‐Specific FFAR4 Deletion Aggravated Tubular Senescence in Fibrotic Kidneys

**FIGURE 4:**
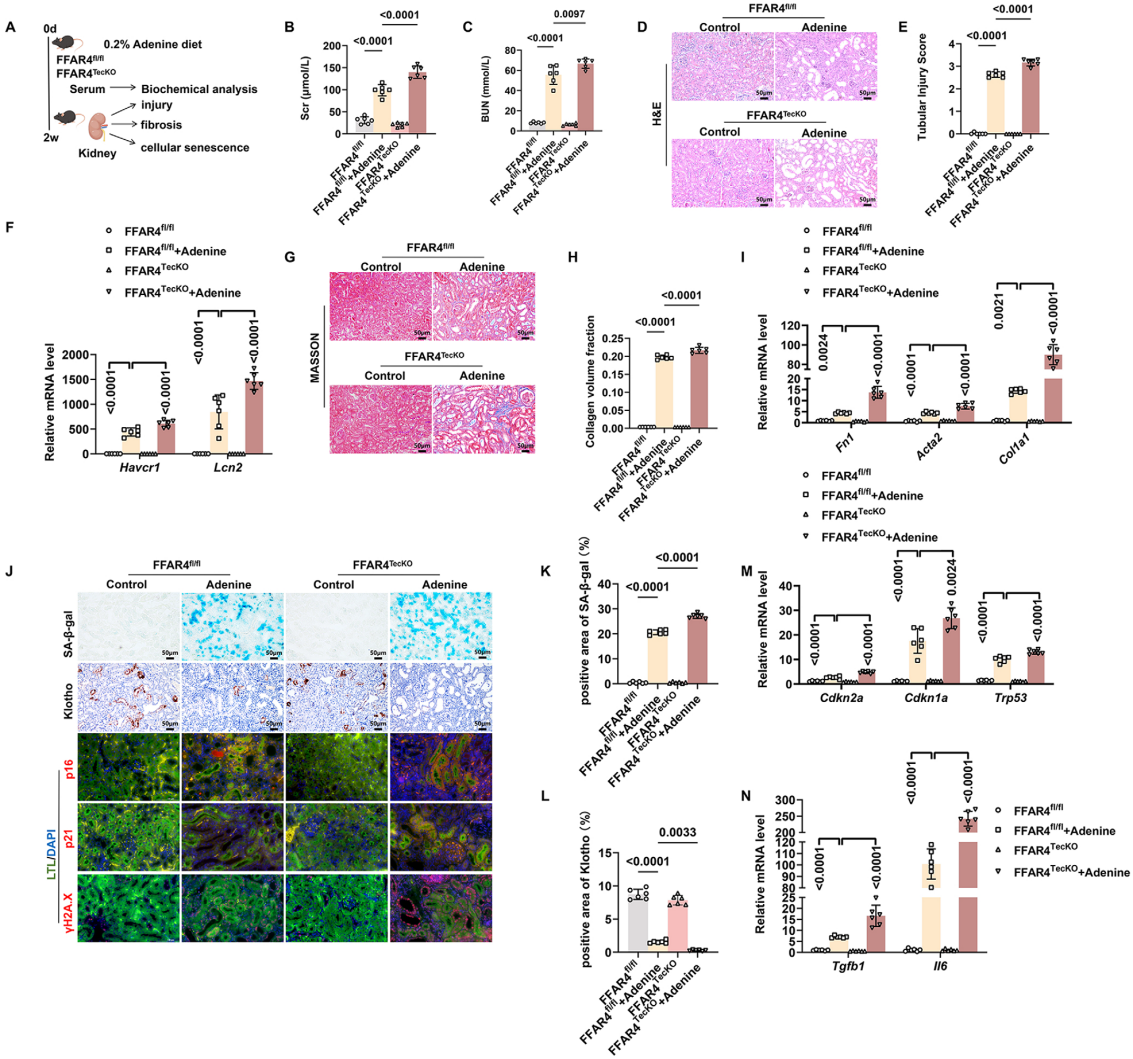
TEC‐specific FFAR4 deletion exacerbated renal fibrosis and tubular senescence in CKD mice. (A) Schematic representation of the animal experiment. FFAR4^TecKO^ mice and FFAR4^fl/fl^ mice fed with 0.2% Adenine diet for 2 weeks. Kidney function, renal pathological injury, tubulointerstitial fibrosis and tubular senescence were assessed at end‐point. (B) Serum creatinine (Scr) of mice (*n* = 6 per group). (C) Blood urea nitrogen (BUN) of mice in each group (*n* = 6 per group). (D) Representative images of H&E staining of kidney sections in each group. (E) Tubular injury scores of kidneys in each group (*n* = 6 per group). (F) Relative mRNA levels of tubular injury markers *Havcr1* and *Lcn2* of kidneys in each group (*n* = 6 per group). (G) Representative images of Masson staining of kidney sections in each group. (H) Collagen volume fraction of kidneys in each group (*n* = 6 per group). (I) Relative mRNA levels of fibrosis‐related genes *Fn1*, *Col1a1*, and *Acta2* in kidneys in each group (*n* = 6 per group). (J) Representative images of SA‐β‐gal staining, immunohistochemical staining of Klotho, double immunofluorescence for p16/p21/γH2A.X and LTL in kidneys in each group. Markers: p16/p21/γH2A.X, red; LTL: green; DAPI, blue. Overlay images (p16/p21 + LTL) appear yellow and purple, due to the colocation of red/green markers and red/blue markers, respectively. Overlay images (γH2A.X + DAPI) appear purple due to the colocation of red and blue markers. (K) Quantification of SA‐β‐gal‐positive areas of kidney sections in each group (*n* = 6 per group). (L) Quantification of immunohistochemical staining of Klotho in kidneys in each group (*n* = 6 per group). (M, N) Relative mRNA levels of senescence‐related genes *Trp53*, *Cdkn1a*, *Cdkn2a*, *Tgfb1*, and *Il6* in kidneys in each group (*n* = 6 per group). Data are presented as mean ± SD. BUN, blood urea nitrogen; Scr, serum creatine.

We further investigated the involvement of tubular FFAR4 in CKD. First, we utilized FFAR4^TecKO^ mice to construct an adenine diet‐induced CKD model (Figure 4A). Compared with FFAR4^fl/fl^ mice, adenine‐induced increases of Scr and BUN were much greater in FFAR4^TecKO^ mice (Figure 4B,C). Meanwhile, TEC‐specific deletion of FFAR4 resulted in more severe tubular injury in adenine diet‐induced mice, characterized by higher mRNA levels of *Havcr1* and *Lcn2*, and worse tubular pathological injury (Figure 4D–F). Furthermore, compared with the FFAR4^fl/fl^ + Adenine group, aggravated renal fibrosis and tubular senescence were observed in the FFAR4^TecKO^ + Adenine group (Figure 4G–N, Figure S6). Similarly, FFAR4 KO exacerbated tubular injury, renal fibrosis, and cellular senescence of TECs in adenine diet‐induced mice (Figures S7 and S8). Furthermore, the similar roles of tubular FFAR4 were illustrated in UUO mice (Figure S9).

### 3.6 FFAR4 Agonism Suppressed Senescent TEC‐Driven Fibroblast Activation via Paracrine Effects

**FIGURE 5:**
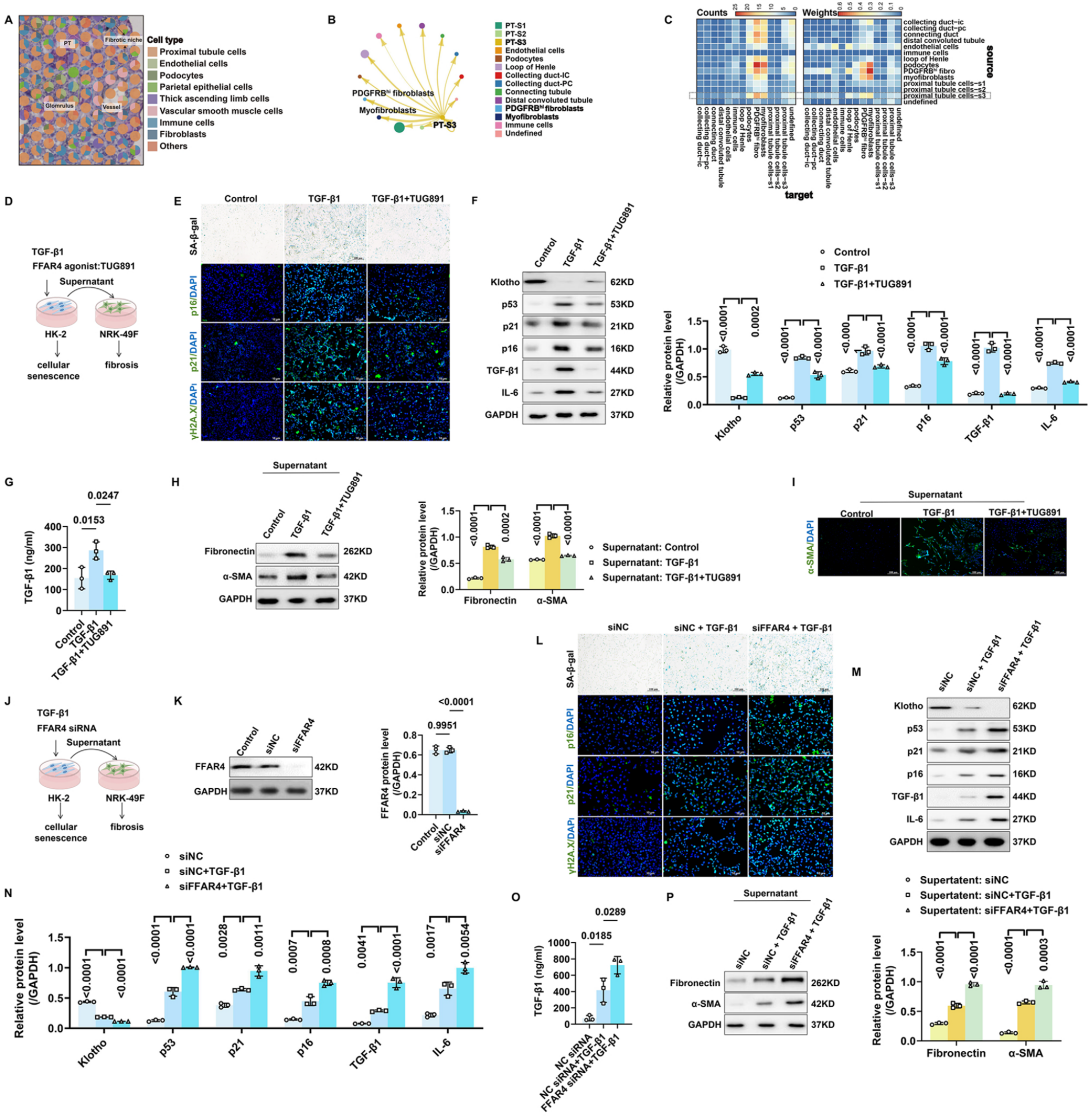
FFAR4 regulated the paracrine effects of senescent TECs on fibroblast activation. (A) Spatial location of each cell type in one representative field of renal cortex from a CKD patient. Yellow dashed areas, consisting of TECs and fibroblasts, indicated fibrotic niche. Pink dashed areas indicated proximal tubule cells. Green dashed areas indicated glomerulus. Blue dashed areas indicated vessels. (B) The circle plot of cell–cell interaction from proximal tubule cell segment 3 to other cell types in the kidneys of patients with CKD. The cell communication results were based on the single‐cell RNA sequencing data from GSE151302. (C) The heatmaps of cell–cell interaction counts and weights between all cell types in the kidneys of patients with CKD. (D) Schematic illustration of the assessment of the paracrine effects of senescent TECs on fibroblast activation. HK‐2 cells were subjected to 10 ng/mL TGF‐β1 treatment for 48 h, simultaneously intervened with TUG891 (10 μM). Then the culture media for TGF‐β1‐treated cells were replaced with fresh media free of TGF‐β1, and incubated for 24 h. The supernatant was collected for stimulating NRK‐49F fibroblasts (1 mL supernatant diluted in 9 mL DMEM) for 48 h. (E) Representative images of SA‐β‐gal staining and immunofluorescence staining of p16, p21, and γH2A.X of HK‐2 cells in each group. Markers: p16/p21/γH2A.X, green; DAPI, blue. Overlay images appear cyan due to the colocation of green and blue markers. (F) Western Blot analysis of senescence markers Klotho, p53, p21, p16, IL‐6, and TGF‐β1 protein expressions in TGF‐β1‐stimulated HK‐2 cells in each group (*n* = 3 per group). (G) Contents of secreted TGF‐β1 by exogenous TGF‐β1‐stimulated HK‐2 cells in each group (*n* = 3 per group), measured by ELISA. (H) Western Blot analysis of fibroblast activation markers Fibronectin and α‐SMA protein expressions in NRK‐49F fibroblasts stimulated with supernatant of HK‐2 cells (*n* = 3 per group). (I) Representative images of immunofluorescence staining of α‐SMA in NRK‐49F fibroblasts stimulated with supernatant of HK‐2 cells. Markers: α‐SMA, green; DAPI, blue. Overlay images appear cyan due to the colocation of green and blue markers. (J) Schematic illustration of the assessment of the paracrine effects of senescent TECs on fibroblast activation. HK‐2 cells were transfected with FFAR4 siRNA, then subjected to 10 ng/mL TGF‐β1 treatment for 48 h. Afterwards, the culture media for TGF‐β1‐treated cells were replaced with fresh media free of TGF‐β1, and incubated for 24 h. The supernatant was collected for stimulating NRK‐49F fibroblasts (1 mL supernatant diluted in 9 mL DMEM) for 48 h. (K) Western Blot analysis of FFAR4 protein expressions in HK‐2 cells transfected with FFAR4 siRNA (*n* = 3 per group). (L) Representative images of SA‐β‐gal staining and immunofluorescence staining of p16, p21, and γH2A.X in HK‐2 cells. Markers: p16/p21/γH2A.X, green; DAPI, blue. Overlay images appear cyan due to the colocation of green and blue markers. (M, N) Western Blot analysis of senescence markers of Klotho, p53, p21, p16, IL‐6, and TGF‐β1 protein expressions in TGF‐β1‐stimulated HK‐2 cells (*n* = 3 per group). (O) Contents of secreted TGF‐β1 by exogenous TGF‐β1‐stimulated HK‐2 cells (*n* = 3 per group) measured by ELISA. (P) Western Blot analysis of fibroblast activation markers of Fibronectin and α‐SMA protein expressions in NRK‐49F fibroblasts stimulated with supernatant of HK‐2 cells. Data are presented as mean ± SD.

As described above, senescent TECs would release multiple pro‐fibrotic factors to activate fibroblasts by paracrine mechanisms (Li et al. 2022, 2023). Herein, we tried to validate this hypothesis via analyzing the public kidney spatial transcriptomic data. The fibrotic niche, consisting of TECs, fibroblasts and other cell types, was evident in kidneys from CKD patients, indicating the absence of crosstalk between TECs and fibroblasts in renal fibrosis progression (Figure 5A). Besides, we further investigated the cell communication among kidney cell types through reanalyzing the single‐cell RNA sequencing (scRNA‐seq) data from human kidney biopsies isolated from DN (the major cause of CKD worldwide) patients (GSE151302, https://doi.org/10.1038/s41467‐022‐32972‐z). Among all other cell types, *PDGFRB* ^*hi*^ fibroblasts and *ACTA2* ^*hi*^ myofibroblasts were the most responsive to secretory signaling from proximal tubule cells, especially the segment 3 (Figure 5B,C) in the kidneys of DN patients. Together, the spatial transcriptome and scRNA‐seq indicated a strong cell–cell interaction between the proximal tubule cells and fibroblasts/myofibroblasts in CKD, largely through the paracrine pathway. Afterwards, we assessed the effects of activating FFAR4 on this crosstalk in vitro. FFAR4 agonist TUG891 was used to intervene TGF‐β1‐stimulated proximal tubular HK‐2 cells, then supernatant from three groups (control, TGF‐β1, and TGF‐β1 + TUG891) was collected to treat NRK‐49F fibroblasts (Figure 5D). As indicated in Figure 5E,F, TGF‐β1‐stimulated HK‐2 cells presented with obvious senescence phenotypes, characterized by increased numbers of SA‐β‐gal‐positive cells, up‐regulation of p53, p16, p21, γH2A.X, TGF‐β1, and IL‐6, and down‐regulation of Klotho. TUG891 treatment showed great anti‐senescence effects. Furthermore, the secretion of TGF‐β1 by HK‐2 cells was measured by ELISA. The results revealed that exogenous stimulation of TGF‐β1 promoted endogenous TGF‐β1 secretion by HK‐2 cells, while such promoting effects were attenuated after TUG891 treatment (Figure 5G).

Next, we assessed the profibrotic effects of supernatant from HK‐2 cells on NRK‐49F fibroblasts activation. NRK‐49F fibroblasts were evidently activated by supernatant from TGF‐β1‐stimulated HK‐2 cells, presented with up‐regulation of α‐SMA and fibronectin. Remarkably, NRK‐49F fibroblasts' activation induced by supernatant from TUG891‐intervened HK‐2 cells was significantly diminished, suggesting inhibition of cellular senescence by activating FFAR4 alleviated the paracrine effects of senescent TECs on fibroblast activation (Figure 5H,I).

To further validate the effects of FFAR4 agonism on improving tubular senescence, we treated HK‐2 cells with H_2_O_2_. As shown in Figure S10, H_2_O_2_ stimulation induced a significant senescent phenotype in HK‐2 cells, characterized by an increased number of SA‐β‐gal‐positive cells, upregulation of p53, p16, IL‐6, and TGF‐β1, and downregulation of Klotho. Treatment with TUG891 alleviated these senescence‐related changes, further confirming the role of FFAR4 in regulating tubular senescence.

### 3.7 Genetic Knockdown of FFAR4 Enhanced the Paracrine Effects of Senescent TECs on Fibroblast Activation

Furthermore, we transfected HK‐2 cells with siRNA to genetically inhibit the expression of FFAR4, exploring the impacts of FFAR4 inhibition on the crosstalk between senescent TECs and fibroblasts (Figure 5J,K). FFAR4 knockdown (siFFAR4) aggravated TGF‐β1‐stimulated cellular senescence of HK‐2 cells (Figure 5L–N). Additionally, after the exogenous stimulation of TGF‐β1, the FFAR4 knockdown group secreted more endogenous TGF‐β1 compared with the scramble siRNA (siNC) group (Figure 5O). Similarly, compared with the supernatant from the siNC + TGF‐β1 group, the supernatant from the siFFAR4 + TGF‐β1 group further promoted the activation of NRK‐49F fibroblasts (Figure 5P and Figure S11).

### 3.8 FFAR4 Regulated Tubular Senescence via PPARγ‐Klotho Signaling in Renal Aging and CKD

**FIGURE 6:**
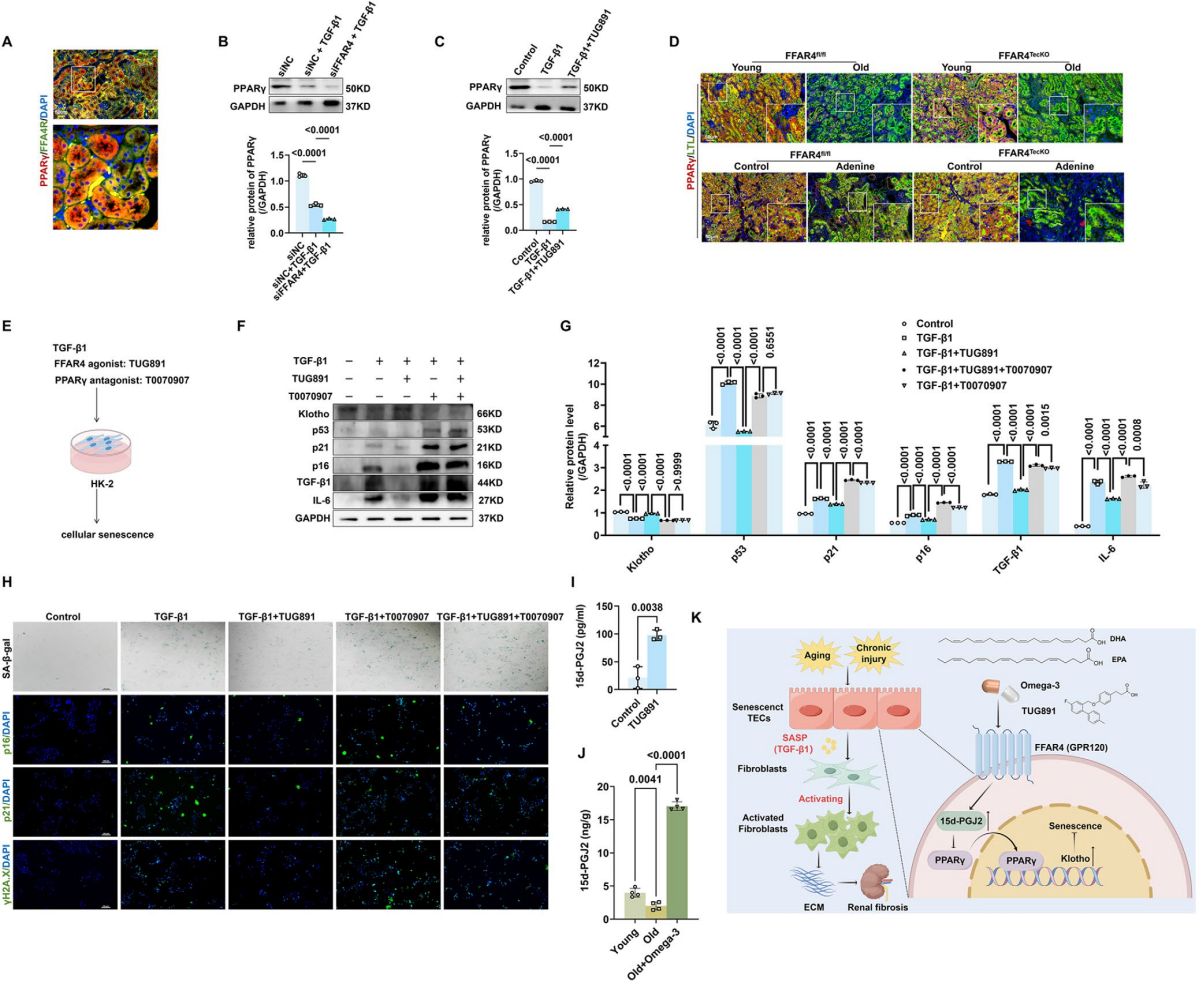
FFAR4 improved tubular senescence by PPARγ‐Klotho signaling in renal aging and CKD. (A) Representative images of double immunofluorescence for FFAR4 and PPARγ in kidneys of a young healthy WT mouse. Markers: PPARγ, red; FFAR4: green; DAPI, blue. Overlay images (FFAR4+ PPARγ) appear yellow due to the colocation of red and green markers. (B, C) Protein expressions of PPARγ in TGF‐β1‐stimulated HK‐2 cells (*n* = 3 per group). (D) Representative images of double immunofluorescence for PPARγ and LTL in kidneys of mice. Markers: PPARγ, red; LTL: green; DAPI, blue. Overlay images (PPARγ+ DAPI) appear purple due to the colocation of red and blue markers. (E) Schematic illustration of the experimental strategy. HK‐2 cells were subjected to 10 ng/mL TGF‐β1 treatment for 48 h, simultaneously intervened with TUG891 (10 μM) and the PPARγ antagonist T0070907 (10 μM), then collected for assessing cellular senescence. (F, G) Westen Blot analysis of senescence markers of Klotho, p53, p21, p16, TGF‐β1, and IL‐6 protein expressions in HK‐2 cells (*n* = 3 per group). (H) Representative images of SA‐β‐gal staining and immunofluorescence staining of p16, p21, and γH2A.X in TGF‐β1‐stimulated HK‐2 cells. Markers: p16/p21/γH2A.X, green; DAPI, blue. Overlay images appear cyan due to the colocation of green and blue markers. (I) Contents of 15d‐PGJ2 in the supernatant of HK‐2 cells measured by ELISA. (J) Contents of 15d‐PGJ2 in kidneys of each group measured by HPLC‐MS/MS. (K) Schematic illustration of omega‐3‐FFAR4 regulating paracrine effects of senescent TECs on fibroblast activation via PPARγ‐Klotho signaling. Data are presented as mean ± SD.

PPARγ is a critical regulator of cellular senescence (Xu et al. 2018, 2020; Sun, Jiang, et al. 2024). Notably, PPARγ is shown to up‐regulate Klotho expression, indicating the potential of PPARγ as a target of renal aging and CKD (Zhang et al. 2008; Oh et al. 2018; Lin et al. 2017; Yang et al. 2009; Zhang and Zheng 2008). Interestingly, FFAR4 could activate PPARγ in adipocytes (Paschoal et al. 2020). Based on these findings, in the present study, we sought to explore whether FFAR4 regulated tubular senescence by PPARγ signaling. Firstly, the colocalization of FFAR4/PPARγ in kidneys was verified by IF staining (Figure 6A). TGF‐β1 also induced a decrease of PPARγ expression in HK‐2 cells, exacerbated by FFAR4 knockdown and reversed by TUG891 treatment (Figure 6B,C). Furthermore, we observed that PPARγ expression was significantly reduced in the kidneys of old mice, adenine diet‐induced and UUO mice. Knockout of FFAR4 further down‐regulated renal PPARγ (Figure 6D, Figures S12 and S13). Simultaneously Co‐IF staining further revealed that the altered PPARγ expressions in kidneys were most evident in TECs (Figure 6D and Figure S13B).

To investigate whether the anti‐senescent effect of FFAR4 occurs via the activation of PPARγ, T0070907 was used to intervene TGF‐β1‐stimulated HK‐2 cells (Figure 6E). Notably, T0070907 reversed the anti‐senescent effect of TUG891. Besides, T0070907 exacerbated TGF‐β1‐induced senescence, unresponsive to TUG891 treatment (Figure 6F–H). The 15d‐PGJ2 is one of the ultimate dehydration products of prostaglandin D2 (PGD2), known as a well‐established endogenous activator of PPARγ. As presented in Figure 6I, TUG891 treatment induced an obvious increase of 15‐d PGJ2 in the supernatant from HK‐2 cells. Moreover, we quantified 15d‐PGJ2 levels in renal tissues of aged mice. Compared with young controls, aged kidneys exhibited a significant reduction in 15‐d‐PGJ_2_. Omega‐3 treatment rescued this decline (Figure 6J). These findings suggest that FFAR4 stimulation activated PPARγ by inducing the endogenous PPARγ ligand 15d‐PGJ2 (Figure 6K).

## 4 Discussion

Renal aging and CKD shared multiple pathophysiological commonalities. The present study illustrated that omega‐3 PUFAs had favorable reno‐protective and anti‐senescent effects both in aged and CKD mice. The expression of tubular FFAR4 abnormally reduced in the elderly and CKD patients, significantly associated with the decline of renal function. TEC‐specific FFAR4 deletion exacerbated renal fibrosis and tubular senescence. These findings expand the understanding of the nephroprotective effects of omega‐3 PUFAs and highlight that the omega‐3 receptor FFAR4 is a potential drug target for renal aging and CKD.

It is well recognized that age‐related structural and morphological changes in the kidney are observed in healthy populations (Yamamoto and Isaka 2024; Kishi et al. 2024). Meanwhile, CKD has been well‐accepted as an age‐related disease and shows characteristics of premature senescence (Yamamoto and Isaka 2024). These facts demonstrate the intimate associations between natural renal aging and CKD, pointing out the feasibility and importance to find common drug targets (Kishi et al. 2024; Denic et al. 2022). However, there were very few relevant research studies. In the current study, age‐related eGFR decline and tubular injury were verified, similar to the renal changes in the CKD group. Additionally, premature tubular senescence was revealed in fibrotic kidneys. Consistent with our findings, Ha, S. et al. also found similar renal changes in 20‐month‐old mice and adenine diet‐induced CKD mice (Ha et al. 2024).

Renal fibrosis is the final common pathway of all types of CKD (Li et al. 2022). In the meantime, tubulointerstitial fibrosis is a robust feature of aged kidneys (Kishi et al. 2024; Xu et al. 2023). Nowadays, inhibiting the abnormal fibroblast activation is the primary focus of intervention for renal fibrosis, and cell–cell communication in renal fibrosis has drawn extensive attention (Li et al. 2022; He et al. 2024). Herein, we found that FFAR4 activation alleviated the paracrine effects of senescent TECs on fibroblast activation, suggesting that targeting the cross‐talk between TECs and fibroblasts might be an efficient therapeutic strategy for renal fibrosis. Similarly, Livingston et al. (2023) found injured TECs could produce pro‐fibrotic factors, leading to fibroblast activation and subsequent renal fibrosis after ischemic acute kidney injury.

Therefore, there is a critical need to find approaches for reducing the secretion of pro‐fibrotic factors of injured TECs. As mentioned above, premature senescence of TECs is an important feature of CKD, which was also confirmed by our study. Notably, SASP is one of the most widely understood characteristics of cellular senescence (O'Reilly et al. 2024; Wang et al. 2024). Several SASP factors have been proved to be pro‐fibrotic, resulting in dysfunction and fibrosis in normal tissues (Wang et al. 2024). In the present study, we illustrated that senescent HK‐2 cells produced TGF‐β1 to activate NRK‐49F fibroblasts, while inhibiting senescence of HK‐2 cells by FFAR4 agonism could significantly reduce the secretion of TGF‐β1. Similar to our findings, Li et al. (2023) demonstrated that an anti‐senescence drug ABT‐263 or knockdown of p16 alleviated the paracrine effects of cisplatin‐stimulated TECs on NRK‐49F fibroblasts.

The above facts imply that targeting tubular senescence represents a therapeutic strategy against renal fibrosis. However, the related researches are very limited. The current study confirmed the anti‐senescence effects of omega‐3 PUFAs and synthetic FFAR4 agonist TUG891. Omega‐3 PUFAs have emerged as a promising therapeutic agent in aging and CKD, with numerous clinical studies focusing on their effects, but relevant conclusions remain inconsistent. In recent years, similar to our study, a growing body of basic researches have sought to elucidate the underlying mechanisms through which omega‐3 PUFAs exert the anti‐senescence effects and ameliorate CKD progression (Xiong et al. 2024; Perazza et al. 2021; Khan et al. 2012; Son et al. 2021). However, researches on omega‐3 receptors remain limited. In this study, we not only characterized the expression and localization of FFAR4 in renal aging and CKD, but also employed TEC‐specific FFAR4 deletion mice to demonstrate its functional significance in regulating tubular senescence and renal fibrosis.

Nowadays, anti‐senescence drugs are classified into senolytics and senomorphics. The senolytic drugs induce the apoptosis of senescent cells, while the senomorphic drugs alleviate damages caused by SASP rather than directly killing senescent cells (Huang et al. 2022; O'Reilly et al. 2024). Herein, we observed that activating FFAR4 obviously reduced the TGF‐β1 secretion of senescent TECs. Thus, omega‐3 PUFAs and synthetic FFAR4 agonists could be considered as senomorphic drugs for renal senescence.

It is really interesting to explore how FFAR4 regulates tubular senescence in aging and CKD. PPARγ plays a key role in various physiological processes including lipid metabolism, insulin sensitivity, energy balance, inflammation regulation, and cell differentiation (Chen et al. 2023; Rui et al. 2025). Recently, the roles of PPARγ in cellular senescence have attracted increasing interests (Sun, Jiang, et al. 2024). Xu et al. (2018, 2020) found that PPARγ ligand rosiglitazone alleviated age‐related metabolic dysfunction and was associated with lifespan extension. Ye et al. (2024) demonstrated that PPARγ activation ameliorated cellular senescence of intestinal stem cells and improved age‐related intestinal dysfunction. Moreover, several studies have confirmed that targeting PPARγ significantly alleviated vascular aging (Sun, Wu, et al. 2024; De Silva et al. 2018). Interestingly, PPARγ is known as a positive regulator of Klotho in kidneys, revealing its therapeutic potential against tubular senescence. PPARγ is a ligand‐activated transcription factor. Several unsaturated fatty acids have been reported to be endogenous activators of PPARγ. Among them, 15d‐PGJ2 is most intensively studied. In the present study, we found that FFAR4 agonists restored tubular PPARγ expression by producing 15d‐PGJ2, similar to the previous findings observed in adipocytes (Paschoal et al. 2020), which partially explained the mechanisms by which FFAR4 agonism improved tubular senescence in aging and CKD.

There are still several limitations in this study. First, it is noteworthy that the pathophysiological features of CKD in the elderly are different with those in the younger group. In the future, we will construct CKD models with aged mice to explore the treatment of CKD in the older population. Second, age subgroups were not analyzed in the naturally aging model. Collectively, our study highlights that agonism of FFAR4 improves renal dysfunction and tubulointerstitial fibrosis in aging and CKD. Targeting of tubular omega‐3 fatty acid receptor FFAR4 may provide a novel approach for aging and CKD.

## Author Contributions

L.M. and L.T. designed experiments. L.Y., L.T., J.L., D.L., C.H., F.G., and R.H. performed experiments. L.Y., L.T., J.L., L.L., and L.M. analyzed the data. L.Y., L.T., J.L., L.L., R.H., P.F., and L.M. wrote the draft of the manuscript, and edited it. All authors have read and approved the article.

## Funding

This work was supported by the National Natural Science Foundation of China (82400794, 82370737, and 82200973), the Sichuan Science and Technology Program (2025ZNSFSC1601), the Postdoctor Research Fund from West China Hospital of Sichuan University (2024HXBH158), the Postdoctoral Fellowship Program of CPSF (GZB20240494), and the 1.3.5 project for disciplines of excellence from West China Hospital of Sichuan University (ZYGD23015).

## Conflicts of Interest

The authors declare no conflicts of interest.

## Supporting information


**Figure S1:** Omega‐3 PUFAs attenuated aging and CKD. Current clinical evidence demonstrated the multifaceted benefits of omega‐3 PUFAs in aging and CKD. In older adults, omega‐3 PUFAs supplementation inhibit telomere shortening, attenuate biological age (measured by DNA methylation‐based epigenetic clocks), mitigate inflammaging, and ameliorate age‐related disorders including cognitive impairment, cardiovascular diseases, and sarcopenia. Meanwhile, in CKD patients, omega‐3 PUFAs supplementation ameliorate lipid metabolic disorders, reduce systemic inflammation, mitigate complications such as cardiovascular diseases and pruritus, and delay CKD progression to end‐stage renal disease (ESRD).
**Figure S2:** Omega‐3 PUFAs alleviated tubular injury and senescence in adenine‐induced fibrotic kidneys. (A) Schematic representation of the animal experiment. Wild‐type mice were fed with 0.2% Adenine diet, simultaneously intervened with omega‐3 PUFAs (2 g/kg/day, p.o.) for 2 weeks (*n* = 6 per group). Kidney function, renal pathological injury, tubulointerstitial fibrosis and tubular senescence were assessed at end‐point. (B) Serum creatinine (Scr) of mice in each group (*n* = 6 per group). (C) Blood urea nitrogen (BUN) of mice in each group (*n* = 6 per group). (D) Relative mRNA levels of tubular injury markers hepatitis A virus cellular receptor 1 (*Havcr1*) and lipocalin‐2 (*Lcn2*) of kidneys in each group (*n* = 6 per group). (E) Representative images of H&E staining and Masson staining of kidney sections in each group. (F) Tubular injury scores of kidneys in each group (*n* = 6 per group). (G) Collagen volume fraction of kidneys in each group (*n* = 6 per group). (H) Relative mRNA levels of fibrosis‐related genes *Acta2*, *Fn1*, *Col6a1*, and *Col1a1* of kidneys in each group (*n* = 6 per group). (I) Representative images of SA‐β‐gal staining, immunohistochemical staining of Klotho, double immunofluorescence for p16/p21/γH2A.X and LTL of kidney sections in each group. Markers: p16/p21/γH2A.X, red; LTL: green; DAPI, blue. Overlay images (p16/p21 + LTL) appear yellow and purple, due to the colocation of red/green markers and red/blue markers, respectively. Overlay images (γH2A.X + DAPI) appear purple due to the colocation of red and blue markers. (J) Quantification of SA‐β‐gal‐positive areas of kidney sections in each group (*n* = 6 per group). (K) Quantification of immunohistochemical staining of Klotho in kidneys in each group (*n* = 6 per group). Data are presented as mean ± SD. BUN, blood urea nitrogen; Scr, serum creatine.
**Figure S3:** Omega‐3 PUFAs alleviated exacerbated renal fibrosis and tubular senescence in UUO mice. (A) Schematic representation of the animal experiment. Wild‐type mice were subjected to undergo UUO surgery, then intervened with omega‐3 PUFAs (2 g/kg/day, p.o.) for 7 days (*n* = 6 per group). Renal pathological injury, tubulointerstitial fibrosis and tubular senescence were assessed at end‐point. (B) Representative images of H&E staining and Masson staining of kidney sections in each group. (C) Tubular injury scores of kidneys in each group (*n* = 6 per group). (D) Collagen volume fraction of kidneys in each group (*n* = 6 per group). (E) Representative images of SA‐β‐gal staining and immunohistochemical staining of Klotho of kidney sections in each group. (F) Quantification of SA‐β‐gal‐positive areas of kidney sections in each group (*n* = 6 per group). (G) Quantification of immunohistochemical staining of Klotho in kidneys of mice in each group (*n* = 6 per group). Data are presented as mean ± SD.
**Figure S4:** TEC‐specific FFAR4 deletion exacerbated renal tubular injury in aged mice. Relative mRNA expression of *Havcr1* and *Lcn2* in kidneys in each group (*n* = 6 per group). Data are presented as mean ± SD.
**Figure S5:** TEC‐specific FFAR4 deletion exacerbated renal tubular senescence in aged mice. Representative images of lipofuscin staining of kidney sections in each group.
**Figure S6:** TEC‐specific FFAR4 deletion exacerbated adenine diet‐induced tubular senescence in mice. Western Blot analysis of senescence markers Klotho, p53, p21, p16, TGF‐β1, and IL‐6 protein expressions in kidneys in each group (*n* = 6 per group).
**Figure S7:** Systemic deletion of FFAR4 exacerbated renal dysfunction, tubulointerstitial fibrosis and tubular senescence in adenine diet‐induced CKD mice. (A) Schematic representation of the animal experiment. FFAR4^−/−^ mice and WT mice fed with 0.2% Adenine diet for 2 weeks. Kidney function, renal pathological injury and tubulointerstitial fibrosis were assessed at end‐point. (B) Serum creatinine (Scr) of mice in each group (*n* = 6 per group). (C) Blood urea nitrogen (BUN) of mice in each group (*n* = 6 per group). (D) Representative images of H&E staining, Masson staining and immunohistochemical staining of α‐SMA of kidney sections in each group. (E) Tubular injury scores of kidneys in each group (*n* = 6 per group). (F) Collagen volume fraction of kidneys in each group (*n* = 6 per group). (G) Quantification of immunohistochemical staining of α‐SMA in kidneys of mice (*n* = 6 per group). (H) Relative mRNA expression of *Havcr1* and *Lcn2* in kidneys in each group (*n* = 6 per group). (I) Relative mRNA expression of *Acta2*, *Fn1*, *Col6a1*, and *Col1a1* in kidneys in each group (*n* = 6 per group). (J) Protein expressions of Fibronectin, Collagen I, Collagen VI and α‐SMA in kidneys in each group (*n* = 6 per group). Data are presented as mean ± SD. BUN, blood urea nitrogen; Scr, serum creatine.
**Figure S8:** Systemic deletion of FFAR4 exacerbated tubular senescence in adenine diet‐induced CKD mice. (A) Schematic representation of the animal experiment. FFAR4^−/−^ mice and WT mice fed with 0.2% Adenine diet for 2 weeks. At end‐point, kidney samples in each group were collected for RNA‐sequencing and tubular senescence was assessed. (B) Representative heatmap of differentially expressed genes in the kidneys of each group (WT: *n* = 3; WT + Adenine: *n* = 4; FFAR4^−/−^: *n* = 3; FFAR4^−/−^ + Adenine: *n* = 4). (C) Comparable analysis between FFAR4^−/−^ + Adenine and WT + Adenine group using KEGG database. (D) GSEA enrichment analysis between FFAR4^−/−^ + Adenine and WT + Adenine group. (E) Representative images of SA‐β‐gal staining and immunohistochemical staining of Klotho of kidney sections in each group. (F) Quantification of SA‐β‐gal‐positive areas of kidney sections in each group (*n* = 6 per group). (G) Quantification of immunohistochemical staining of Klotho in kidneys in each group (*n* = 6 per group). (H) Relative mRNA expression of *Kl*, *Trp53*, *Cdkn2a*, and *Cdkn1a* in kidneys in each group (*n* = 6 per group). (I) Relative mRNA expression of *Tgfb1* and *Il6* in kidneys in each group (*n* = 6 per group). (J, K) Protein expressions of Klotho, p53, p21, p16, TGF‐β1and IL‐6 in kidneys in each group (*n* = 6 per group). Data are presented as mean ± SD.
**Figure S9:** Systemic deletion of FFAR4 exacerbated renal fibrosis and tubular senescence in UUO mice. (A) Schematic representation of the animal experiment. FFAR4^−/−^ mice and WT mice were subjected to undergo UUO surgery. Renal pathological injury, tubulointerstitial fibrosis and tubular senescence were assessed at end‐point. (B) Representative images of H&E staining and Masson staining of kidney sections in each group. (C) Tubular injury scores of kidneys in each group (*n* = 6). (D) Collagen volume fraction of kidneys in each group (*n* = 6 per group). (E) Relative mRNA expression of *Havcr1* and *Lcn2* in kidneys in each group (*n* = 6 per group). (F) Relative mRNA expression of *Acta2*, *Fn1*, *Col6a1*, and *Col1a1* in kidneys in each group (*n* = 6 per group). (G, H) Protein expressions of Fibronectin, Collagen I, Collagen VI and α‐SMA in kidneys in each group (*n* = 6 per group). (I) Representative images of SA‐β‐gal staining and immunohistochemical staining of Klotho of kidney sections in each group. (J) Quantification of SA‐β‐gal‐positive areas of kidney sections in each group (*n* = 6 per group). (K) Quantification of immunohistochemical staining of Klotho in kidneys in each group (*n* = 6 per group). Data are presented as mean ± SD.
**Figure S10:** FFAR4 activation alleviated hydrogen peroxide‐induced cellular senescence in HK‐2 cells. (A) Schematic illustration of the assessment of TUG891 ameliorating H_2_O_2_‐induced tubular senescence. HK‐2 cells were stimulated with 400 μmol/L H_2_O_2_ for 2 h, followed by medium replacement and continued culture for 72 h, with TUG891 treatment administered throughout the entire period. (B) Representative images of SA‐β‐gal staining of HK‐2 cells in each group. (C) Western Blot analysis of senescence markers Klotho, p53, p16, TGF‐β1, and IL‐6 protein expressions in H_2_O_2_‐stimulated HK‐2 cells in each group (*n* = 3 per group). Data are presented as mean ± SD.
**Figure S11:** FFAR4 silencing enhanced the paracrine effects of senescent TECs on fibroblast activation. Representative images of immunofluorescence staining of α‐SMA in NRK‐49F fibroblasts stimulated with supernatant of HK‐2 cells. Markers: α‐SMA, green; DAPI, blue. Overlay images appear cyan due to the colocation of green and blue markers. Data are presented as mean ± SD.
**Figure S12:** FFAR4 regulated expressions of PPARγ in kidneys of adenine diet‐induced CKD mice. Protein expressions of PPARγ in kidneys in each group (*n* = 6 per group). Data are presented as mean ± SD.
**Figure S13:** FFAR4 regulated expressions of PPARγ in kidneys of UUO mice. (A) Protein expressions of PPARγ in kidneys in each group (*n* = 6 per group). (B) Representative images of double immunofluorescence for PPARγ and LTL in kidneys of mice. Markers: PPARγ, red; LTL: green; DAPI, blue. Overlay images (PPARγ+ DAPI) appear purple due to the colocation of red and blue markers. Data are presented as mean ± SD.
**Figure S14:** Generation of FFAR4‐KO mice. (A) Schematic representation of FFAR4‐KO mice generation. (B, C) Identification of the genotype of FFAR4‐KO mice by PCR assay.
**Figure S15:** Validation of constitutive FFAR4 Knockout. (A) Relative mRNA levels of FFAR4 of kidneys in each group (*n* = 6 per group). (B) Western Blot analysis of FFAR4 protein expressions of kidneys in each group (*n* = 6 per group). (C) Representative images of double immunofluorescence staining for FFAR4 and LTL in kidneys of WT mice and FFAR4^−/−^ mice. Markers: FFAR4, red; LTL: green; DAPI, blue. Overlay images (FFAR4 + LTL) appear yellow due to the colocation of red and green markers.
**Figure S16:** Generation of renal tubular epithelial cell‐specific (TEC‐specific) FFAR4 KO mice. (A) Schematic representation of FFAR4^flox/flox^ (FFAR4^fl/fl^) mice generation by CRISPR/Cas9‐stimulated homologous recombination and design strategy of TEC‐specific FFAR4 KO (FFAR4^tecKO^) mice. (B, C) Identification of the genotype of FFAR4^fl/fl^ mice and FFAR4^tecKO^ (Cdh16‐Cre+ FFAR4^fl/fl^) mice by PCR assay.
**Figure S17:** Validation of TEC‐specific FFAR4 knockout. (A) Relative mRNA levels of FFAR4 of kidneys in each group (*n* = 6 per group). (B) Western Blot analysis of FFAR4 protein expressions of kidneys in each group (*n* = 6 per group). (C) Representative images of double immunofluorescence staining for FFAR4, LTL, PNA and AQP3 in kidneys of FFAR4^fl/fl^ and FFAR4^TecKO^ mice. Markers: FFAR4, red; LTL, PNA and AQP3: green; DAPI, blue. Overlay images (FFAR4 + LTL/FFAR4 + PNA/FFAR4 + AQP3) appear yellow due to the colocation of red and green markers.
**Table S1:** Clinical characteristics of enrolled old and young subjects.
**Table S2:** Clinical characteristics of enrolled CKD patients and control subjects.
**Table S3:** The list of primary antibodies.
**Table S4:** The list of primer sequences.

## Data Availability

All data supporting the study have been included in this manuscript and its Supporting Information files. Additional data are available from the corresponding author on request.
