# Supplementary material for: Tubular Omega‐3 Fatty Acid Receptor FFAR4 Deficiency Aggravated Renal Aging and Chronic Kidney Disease
Source: Aging Cell. 2026 May 23;25(6):e70546. doi: 10.1111/acel.70546 (PMC13239551; doi:10.1111/acel.70546)
Supplement: Supplementary file 1 — Figure S1: Omega‐3 PUFAs attenuated aging and CKD. Current clinical evidence demonstrated the multifaceted benefits of omega‐3 PUFAs in aging and CKD. In older adults, omega‐3 PUFAs supplementation inhibit telomere shortening, attenuate biological age (measured by DNA methylation‐based epigenetic clocks), mitigate inflammaging, and ameliorate age‐related disorders including cognitive impairment, cardiovascular diseases, and sarcopenia. Meanwhile, in CKD patients, omega‐3 PUFAs supplementation ameliorate lipid metabolic disorders, reduce systemic inflammation, mitigate complications such as cardiovascular diseases and pruritus, and delay CKD progression to end‐stage renal disease (ESRD). Figure S2: Omega‐3 PUFAs alleviated tubular injury and senescence in adenine‐induced fibrotic kidneys. (A) Schematic representation of the animal experiment. Wild‐type mice were fed with 0.2% Adenine diet, simultaneously intervened with omega‐3 PUFAs (2 g/kg/day, p.o.) for 2 weeks (n = 6 per group). Kidney function, renal pathological injury, tubulointerstitial fibrosis and tubular senescence were assessed at end‐point. (B) Serum creatinine (Scr) of mice in each group (n = 6 per group). (C) Blood urea nitrogen (BUN) of mice in each group (n = 6 per group). (D) Relative mRNA levels of tubular injury markers hepatitis A virus cellular receptor 1 (Havcr1) and lipocalin‐2 (Lcn2) of kidneys in each group (n = 6 per group). (E) Representative images of H&E staining and Masson staining of kidney sections in each group. (F) Tubular injury scores of kidneys in each group (n = 6 per group). (G) Collagen volume fraction of kidneys in each group (n = 6 per group). (H) Relative mRNA levels of fibrosis‐related genes Acta2, Fn1, Col6a1, and Col1a1 of kidneys in each group (n = 6 per group). (I) Representative images of SA‐β‐gal staining, immunohistochemical staining of Klotho, double immunofluorescence for p16/p21/γH2A.X and LTL of kidney sections in each group. Markers: p16/p21/γH2A.X, red; LTL: g [file ACEL-25-e70546-s001.pdf]

Supplementary Materials for

Tubular omega-3 fatty acid receptor FFAR4 deficiency aggravated renal aging and chronic kidney disease

Letian Yang [1#], Lei Tang [1#], Jian Li[1#], Dekai Liu[1], Chunchun Hu[1], Fan Guo[1], Lin Lin[2]*, Rongshuang Huang[1]*, Ping Fu[1]*, Liang Ma[1]*

1. Department of Nephrology, Institute of Kidney Diseases, West China Hospital of Sichuan University, Chengdu 610041, China.
2. West-district Outpatient Department, West China Hospital of Stomatology, Sichuan University, Chengdu 610041, China.

[#] These authors contributed equally to this work.

*Correspondence to: Liang Ma, Ping Fu, Rongshuang Huang and Lin Lin. E-Mail: liang_m@scu.edu.cn (Ma L), fupinghx@scu.edu.cn (Fu P), huangrongshuang@wchscu.cn (Huang RS), and linlin_stomatology@foxmail.com (Lin L)

The file includes:

Materials and Methods

Supplementary Figure 1 to 17

Supplementary Table 1-4

Materials and Methods

Animals

The strategies used to generate FFAR4-KO mice and $FFAR4^{TecKO}$ mice have been described previously. FFAR4 wild type (WT) and knockout (KO) mice in C57BL/6J background, $FFAR4^{flox/flox}$ ($FFAR4^{fl/fl}$) and renal tubular epithelial cell-specific (TEC-specific) conditional FFAR4 KO (Cdh16-Cre+$FFAR4^{fl/fl}$, $FFAR4^{TecKO}$) mice in C57BL/6J background were purchased from GemPharmatech, Nanjing, China. FFAR4 KO target site, sequence details and identification of the genotypes of mice were presented in Supplementary Figure 14. The validation of constitutive FFAR4 Knockout was illustrated in Supplementary Figure 15. The construction of $FFAR4^{fl/fl}$ mice is based on CRISPR/Cas9-stimulated homologous recombination. Briefly, exon 2 and exon 3 of the FFAR4 gene were flanked by two LoxP elements. Two heterozygous recombinant embryonic stem cells clones screened by homologous recombination were identified and microinjected into blastocysts from C57BL/6J mice to generate floxed heterozygous mice ($FFAR4^{flox/+}$). $FFAR4^{flox/+}$ mice were then inbred to obtain homozygous FFAR4-floxed mice ($FFAR4^{fl/fl}$). To generate $FFAR4^{TecKO}$ mice, $FFAR4^{f/f}$ mice were crossed with Cdh16-Cre mice. The genotype of $FFAR4^{TecKO}$ mice was confirmed by PCR assay using specific primers (Supplementary Figure 16). Littermates carried the $FFAR4^{fl/fl}$ transgene were used as controls. The validation of TEC-specific FFAR4 knockout was illustrated in Supplementary Figure 17.

Cell transfection with siRNAs

The human FFAR4 siRNA and human negative control (NC) siRNA were purchased from GenePharma (Shanghai, China). HK-2 cells transfection with siRNAs was conducted using Lipofectamine 2000 (12566014, Invitrogen, CA, USA) according to the manufacturer's instructions. The sequences were listed as follows:

FFAR4 siRNA, sense 5′-GCCUUCACAUUUGCUAAUUTT-3′, and antisense 5′-AAUUAGCAAAUGUGAAGGCTT′-3′.

NC siRNA, sense 5′- UUCUCCGAACGUGUCACGUTT, and antisense 5′-ACGUGACACGUUCGGAGAATT-3′.

The measurements of renal 15-deoxy-Δ12,14-Prostaglandin J2

Following gradual thawing at 4°C, kidney aliquots were homogenized in ice-cold methanol/acetonitrile/water (2:2:1, v/v) with vortexing, subjected to 30-min ultrasonication at 4°C, incubated at -20°C for 10 min, and centrifuged (14,000 × g, 4°C, 20 min). The supernatant was lyophilized and reconstituted in 100 μL acetonitrile/water (1:1, v/v), followed by recentrifugation (14,000 × g, 4°C, 15 min). Chromatographic separation was achieved on an Agilent 1290 Infinity LC system using two complementary columns: 1) HILIC column (35°C, 0.3 mL/min) with mobile phase A (water/acetonitrile 90:10 + 2 mM ammonium formate) and B (acetonitrile + 0.4% formic acid) under gradient elution: 0-1.0 min 85% B, 1.0-3.0 min 85→80% B, 3.0-4.0 min 80% B, 4.0-6.0 min 80→70% B, 6.0-10.0 min 70→50% B, 10.0-15.5 min 50% B, 15.5-15.6 min 50→85% B, 15.6-23.0 min 85% B; 2) C18 column (40°C, 0.4 mL/min) with mobile phase A (water + 5 mM ammonium acetate) and B (acetonitrile) using gradient: 0-5.0 min 5→60% B, 5.0-11.0 min 60→100% B, 11.0-13.0 min 100% B, 13.0-13.1 min 100→5% B, 13.1-16.0 min 5% B. Samples were maintained at 4°C in the autosampler and analyzed in randomized order with QC samples inserted every 10 injections. Mass spectrometry was performed on an AB Sciex 6500+ QTRAP instrument operated in MRM mode with ESI source parameters: temperature 580°C, GS1 45 psi, GS2 60 psi, CUR 35 psi, IS ±4500 V. Quantification was achieved via MultiQuant 3.0.3 using analyte/internal standard peak area ratios calibrated against standard curves ($R^2 > 0.995$), with data acceptance requiring QC sample RSD < 15%.

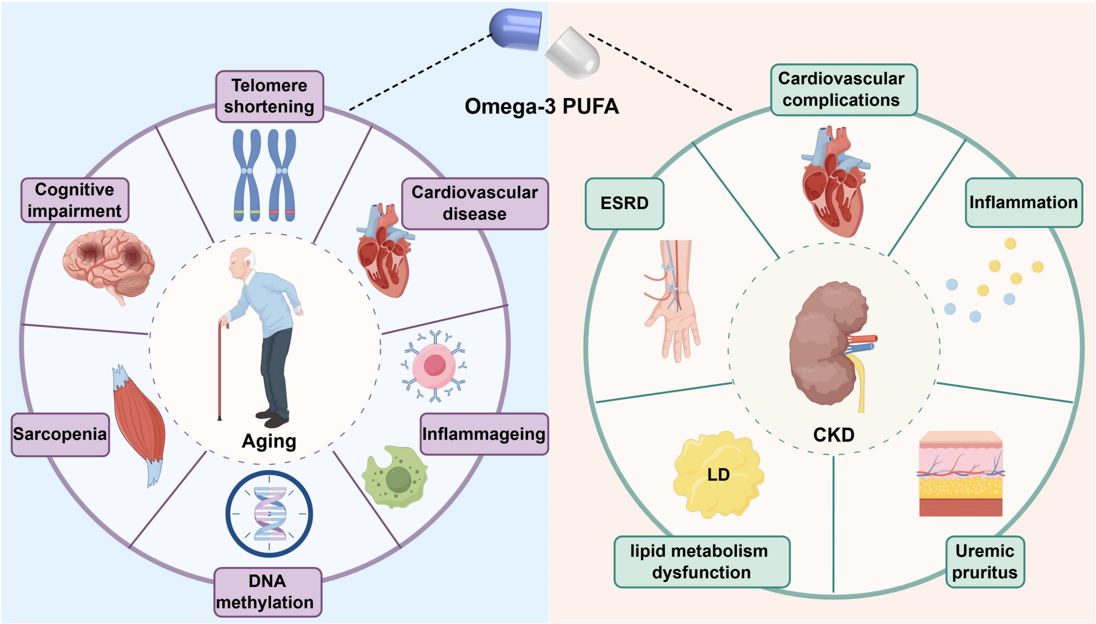

Supplementary Figure 1. Omega-3 PUFAs attenuated aging and CKD. Current clinical evidence demonstrated the multifaceted benefits of omega-3 PUFAs in aging and CKD. In older adults, omega-3 PUFAs supplementation inhibit telomere shortening, attenuate biological age (measured by DNA methylation-based epigenetic clocks), mitigate inflammaging, and ameliorate age-related disorders including cognitive impairment, cardiovascular diseases, and sarcopenia. Meanwhile, in CKD patients, omega-3 PUFAs supplementation ameliorate lipid metabolic disorders, reduce systemic inflammation, mitigate complications such as cardiovascular diseases and pruritus, and delay CKD progression to end-stage renal disease (ESRD).

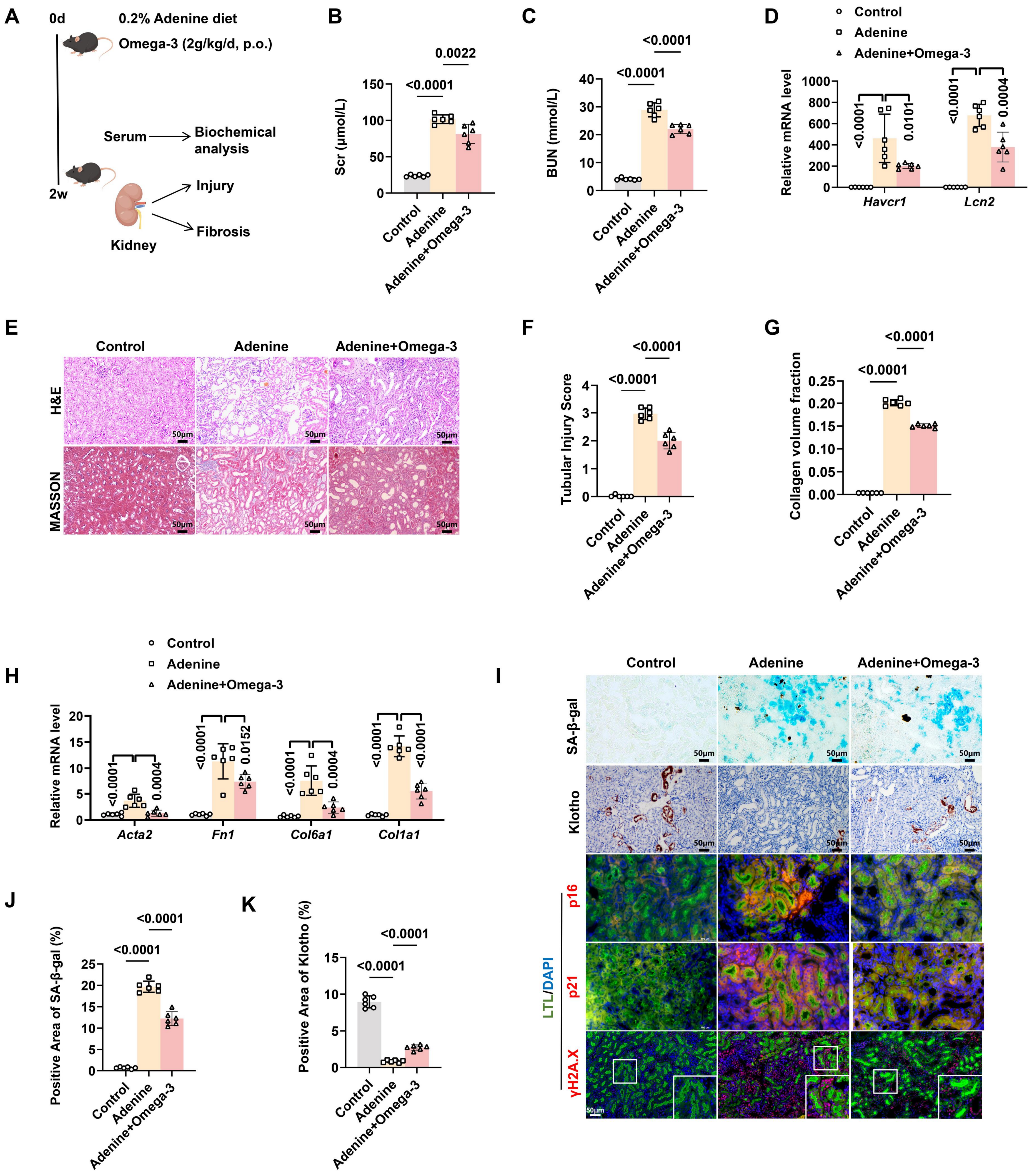

Supplementary Figure 2. Omega-3 PUFAs alleviated tubular injury and senescence in adenine-induced fibrotic kidneys. A Schematic representation of the animal experiment. Wild-type mice were fed with 0.2% Adenine diet, simultaneously intervened with omega-3 PUFAs (2g/kg/d, p.o.) for 2 weeks (n=6 per group). Kidney function, renal pathological injury, tubulointerstitial fibrosis and tubular senescence were assessed at end-point. **B** Serum creatinine (Scr) of mice in each group (n=6 per group). **C** Blood urea nitrogen (BUN) of mice in each group (n=6 per group). **D** Relative mRNA levels of tubular injury markers hepatitis A virus cellular receptor 1 (*Havcr1*) and lipocalin-2 (*Lcn2*) of kidneys in each group (n=6 per group). **E** Representative images of H&E staining and Masson staining of kidney sections in

each group. **F** Tubular injury scores of kidneys in each group (n=6 per group). **G** Collagen volume fraction of kidneys in each group (n=6 per group). **H** Relative mRNA levels of fibrosis-related genes *Acta2*, *Fn1*, *Col6a1* and *Col1a1* of kidneys in each group (n=6 per group). **I** Representative images of SA-β-gal staining, immunohistochemical staining of Klotho, double immunofluorescence for p16/p21/γH2A.X and LTL of kidney sections in each group. Markers: p16/p21/γH2A.X, red; LTL: green; DAPI, blue. Overlay images (p16/p21+LTL) appear yellow and purple, due to the colocation of red/green markers and red/blue markers, respectively. Overlay images (γH2A.X + DAPI) appear purple due to the colocation of red and blue markers. **J** Quantification of SA-β-gal-positive areas of kidney sections in each group (n=6 per group). **K** Quantification of immunohistochemical staining of Klotho in kidneys in each group (n=6 per group). Data are presented as mean ± SD. Scr: serum creatine; BUN: blood urea nitrogen.

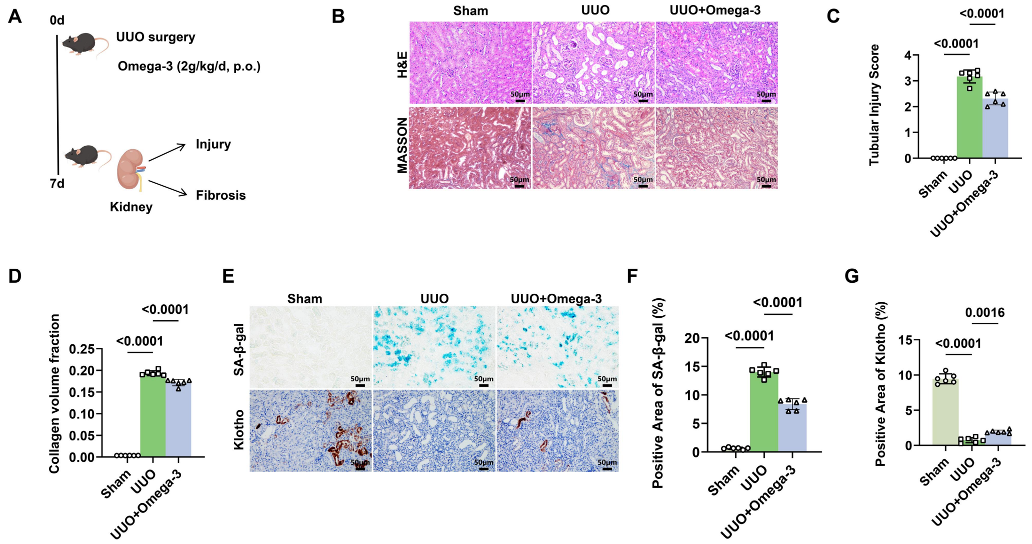

Supplementary Figure 3. Omega-3 PUFAs alleviated exacerbated renal fibrosis and tubular senescence in UUO mice. A Schematic representation of the animal experiment. Wild-type mice were subjected to undergo UUO surgery, then intervened with omega-3 PUFAs (2g/kg/d, p.o.) for 7 days (n=6 per group). Renal pathological injury, tubulointerstitial fibrosis and tubular senescence were assessed at end-point. **B** Representative images of H&E staining and Masson staining of kidney sections in each group. **C** Tubular injury scores of kidneys in each group (n=6 per group). **D** Collagen volume fraction of kidneys in each group (n=6 per group). **E** Representative images of SA-β-gal staining and immunohistochemical staining of Klotho of kidney sections in each group. **F** Quantification of SA-β-gal-positive areas of kidney sections in each group (n=6 per group). **G** Quantification of immunohistochemical staining of Klotho in kidneys of mice in each group (n=6 per group). Data are presented as mean ± SD.

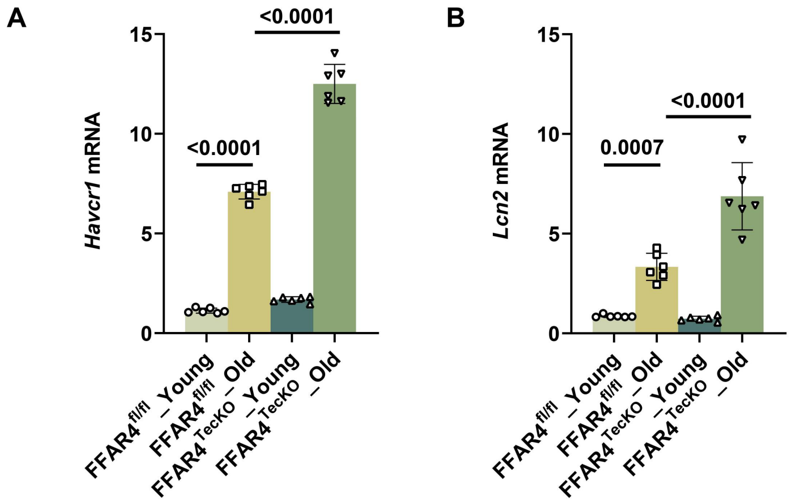

Supplementary Figure 4. TEC-specific FFAR4 deletion exacerbated renal tubular injury in aged mice. Relative mRNA expression of *Havcr1* and *Lcn2* in kidneys in each group (n=6 per group). Data are presented as mean ± SD.

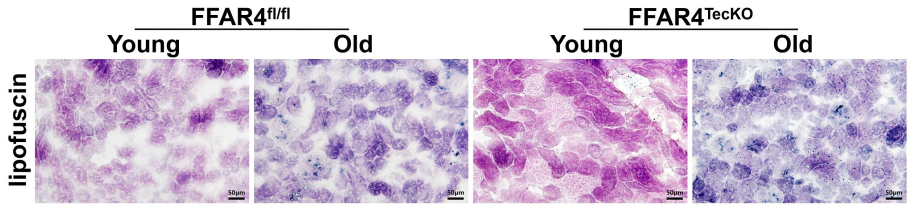

Supplementary Figure 5. TEC-specific FFAR4 deletion exacerbated renal tubular senescence in aged mice. Representative images of lipofuscin staining of kidney sections in each group.

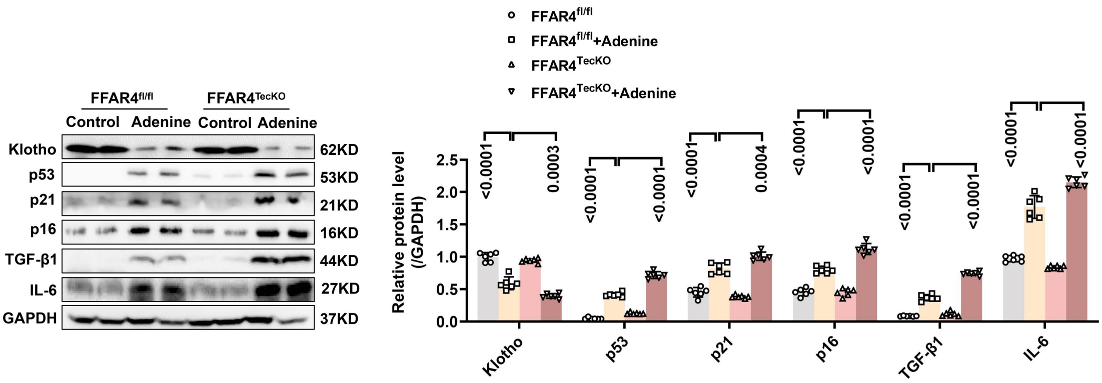

Supplementary Figure 6. TEC-specific FFAR4 deletion exacerbated adenine diet-induced tubular senescence in mice. Western Blot analysis of senescence markers Klotho, p53, p21, p16, TGF-β1 and IL-6 protein expressions in kidneys in each group (n=6 per group).

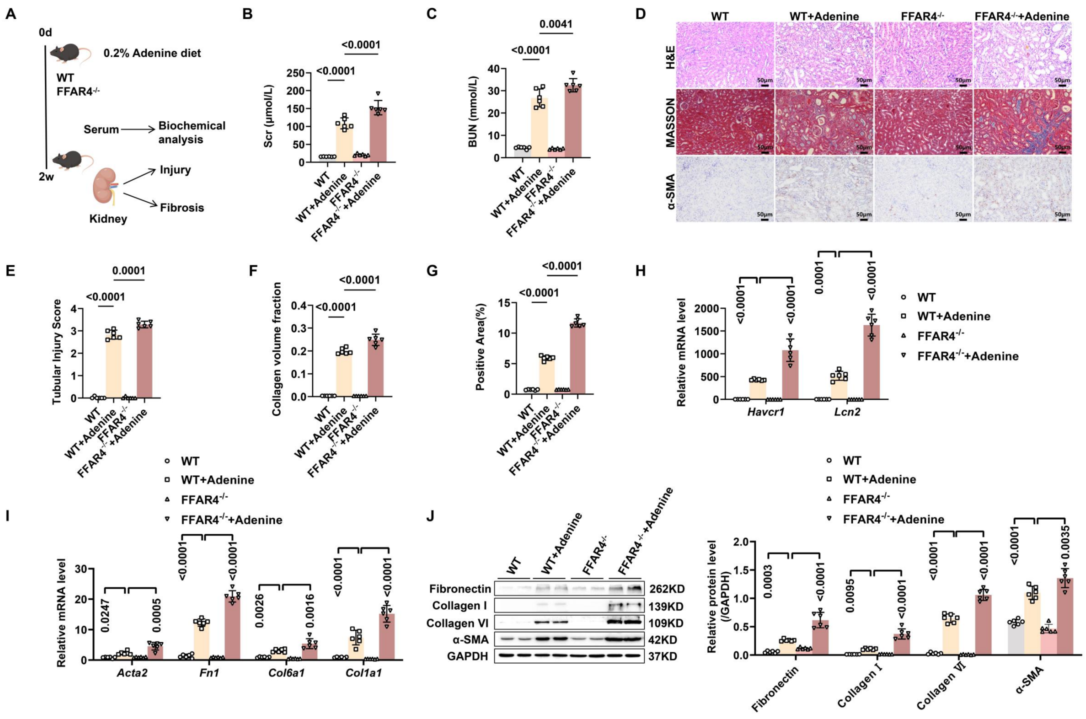

Supplementary Figure 7. Systemic deletion of FFAR4 exacerbated renal dysfunction, tubulointerstitial fibrosis and tubular senescence in adenine diet-induced CKD mice. A Schematic representation of the animal experiment. FFAR4$^{-/-}$ mice and WT mice fed with 0.2% Adenine diet for 2 weeks. Kidney function, renal pathological injury and tubulointerstitial fibrosis were assessed at end-point. **B** Serum creatinine (Scr) of mice in each group (n=6 per group). **C** Blood urea nitrogen (BUN) of mice in each group (n=6 per group). **D** Representative images of H&E staining, Masson staining and immunohistochemical staining of α-SMA of kidney sections in each group. **E** Tubular injury scores of kidneys in each group (n=6 per group). **F** Collagen volume fraction of kidneys in each group (n=6 per group). **G** Quantification of immunohistochemical staining of α-SMA in kidneys of mice (n=6 per group). **H** Relative mRNA expression of *Havcr1* and *Lcn2* in kidneys in each group (n=6 per group). **I** Relative mRNA expression of *Acta2*, *Fn1*, *Col6a1* and *Col1a1* in kidneys in each group (n=6 per group). **J** Protein expressions of Fibronectin, Collagen I, Collagen VI and α-SMA in kidneys in each group (n=6 per group). Data are presented as mean ± SD. Scr: serum creatine; BUN: blood urea nitrogen.

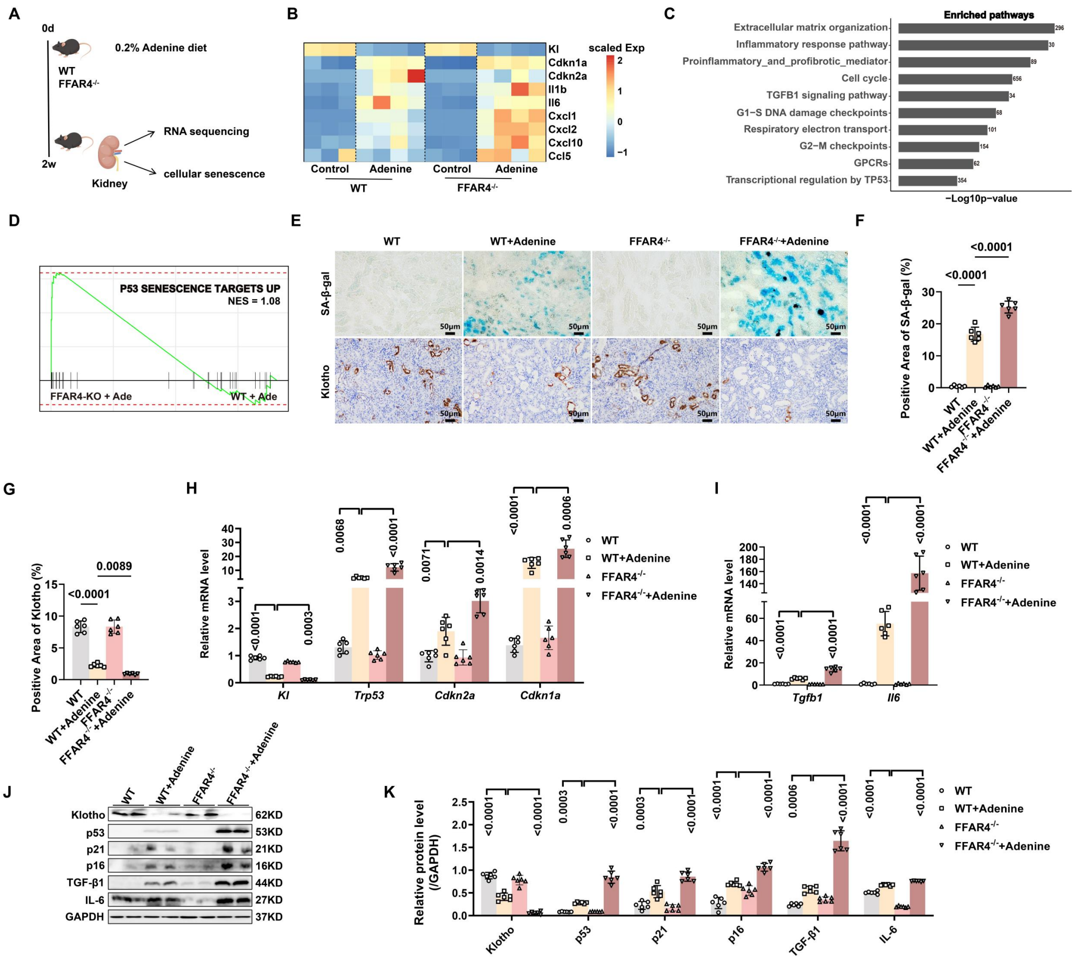

Supplementary Figure 8. Systemic deletion of FFAR4 exacerbated tubular senescence in adenine diet-induced CKD mice. A Schematic representation of the animal experiment. FFAR4$^{-/-}$ mice and WT mice fed with 0.2% Adenine diet for 2 weeks. At end-point, kidney samples in each group were collected for RNA-sequencing and tubular senescence was assessed. **B** Representative heatmap of differentially expressed genes in the kidneys of each group (WT: n=3; WT+Adenine: n=4; FFAR4$^{-/-}$: n=3; FFAR4$^{-/-}$+Adenine: n=4). **C** Comparable analysis between FFAR4$^{-/-}$+Adenine and WT+Adenine group using KEGG database. **D** GSEA enrichment analysis between FFAR4$^{-/-}$+Adenine and WT+Adenine group. **E** Representative images of SA-β-gal staining and immunohistochemical staining of Klotho of kidney sections in each group. **F** Quantification of SA-β-gal-positive areas of kidney sections in each group (n=6 per group). **G** Quantification of immunohistochemical staining of Klotho in kidneys in each group (n=6 per group). **H** Relative mRNA expression of *Kl*, *Trp53*, *Cdkn2a*, and *Cdkn1a* in kidneys in each

group (n=6 per group). **I** Relative mRNA expression of *Tgfb1* and *Il6* in kidneys in each group (n=6 per group). **J-K** Protein expressions of Klotho, p53, p21, p16, TGF-β1and IL-6 in kidneys in each group (n=6 per group). Data are presented as mean ± SD.

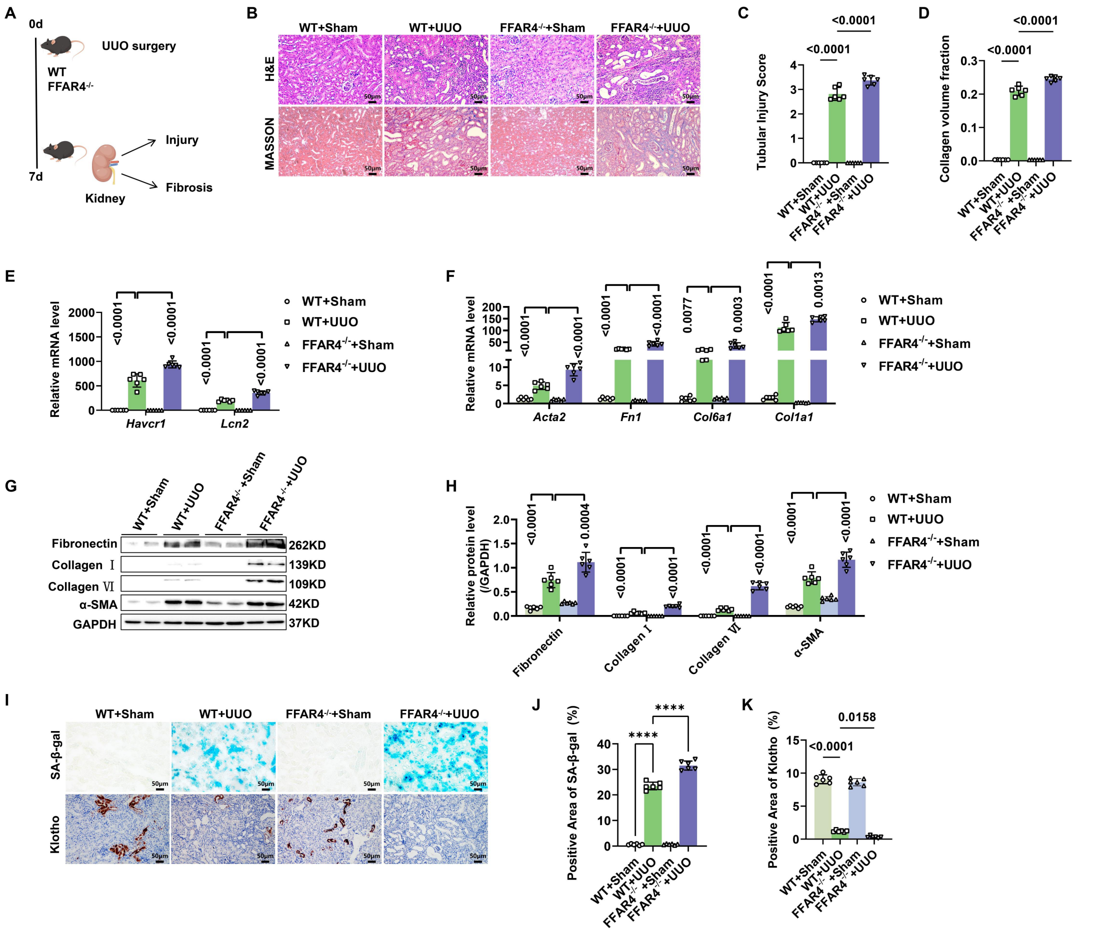

Supplementary Figure 9. Systemic deletion of FFAR4 exacerbated renal fibrosis and tubular senescence in UUO mice. A Schematic representation of the animal experiment. $FFAR4^{-/-}$ mice and WT mice were subjected to undergo UUO surgery. Renal pathological injury, tubulointerstitial fibrosis and tubular senescence were assessed at end-point. **B** Representative images of H&E staining and Masson staining of kidney sections in each group. **C** Tubular injury scores of kidneys in each group (n=6). **D** Collagen volume fraction of kidneys in each group (n=6 per group). **E** Relative mRNA expression of *Havcr1* and *Lcn2* in kidneys in each group (n=6 per group). **F** Relative mRNA expression of *Acta2*, *Fn1*, *Col6a1* and *Col1a1* in kidneys in each group (n=6 per group). **G-H** Protein expressions of Fibronectin, Collagen I, Collagen VI and α-SMA in kidneys in each group (n=6 per group). **I** Representative images of SA-β-gal staining and immunohistochemical staining of Klotho of kidney sections in each group. **J** Quantification of SA-β-gal-positive areas of kidney sections

in each group (n=6 per group). **K** Quantification of immunohistochemical staining of Klotho in kidneys in each group (n=6 per group). Data are presented as mean ± SD.

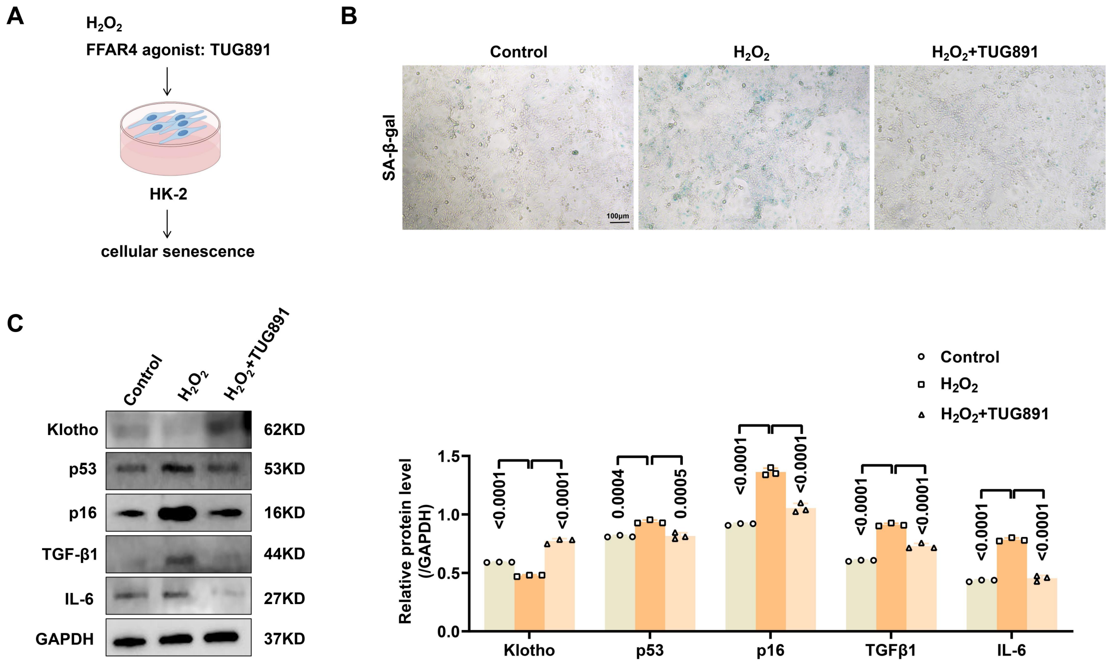

Supplementary Figure 10. FFAR4 activation alleviated hydrogen peroxide-induced cellular senescence in HK-2 cells. **A** Schematic illustration of the assessment of TUG891 ameliorating H_2O_2-induced tubular senescence. HK-2 cells were stimulated with 400 μmol/L H_2O_2 for 2h, followed by medium replacement and continued culture for 72h, with TUG891 treatment administered throughout the entire period. **B** Representative images of SA-β-gal staining of HK-2 cells in each group. **C** Western Blot analysis of senescence markers Klotho, p53, p16, TGF-β1 and IL-6 protein expressions in H_2O_2-stimulated HK-2 cells in each group (n=3 per group). Data are presented as mean ± SD.

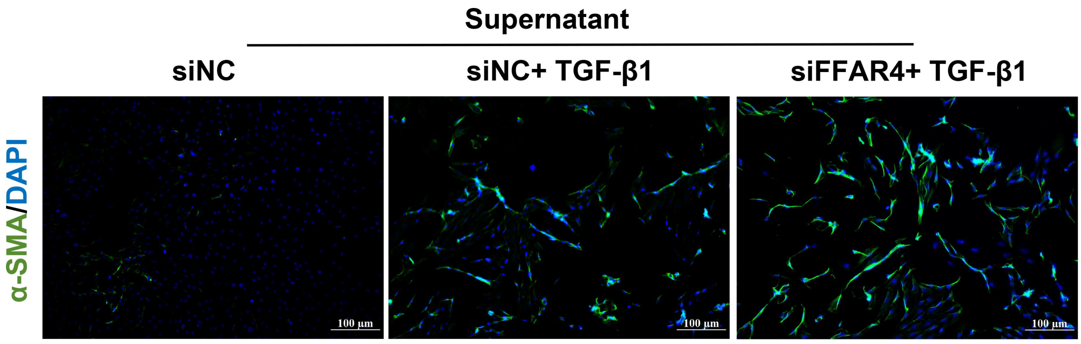

Supplementary Figure 11. FFAR4 silencing enhanced the paracrine effects of senescent TECs on fibroblast activation. Representative images of immunofluorescence staining of α-SMA in NRK-49F fibroblasts stimulated with supernatant of HK-2 cells. Markers: α-SMA, green; DAPI, blue. Overlay images appear cyan due to the colocation of green and blue markers. Data are presented as mean ± SD.

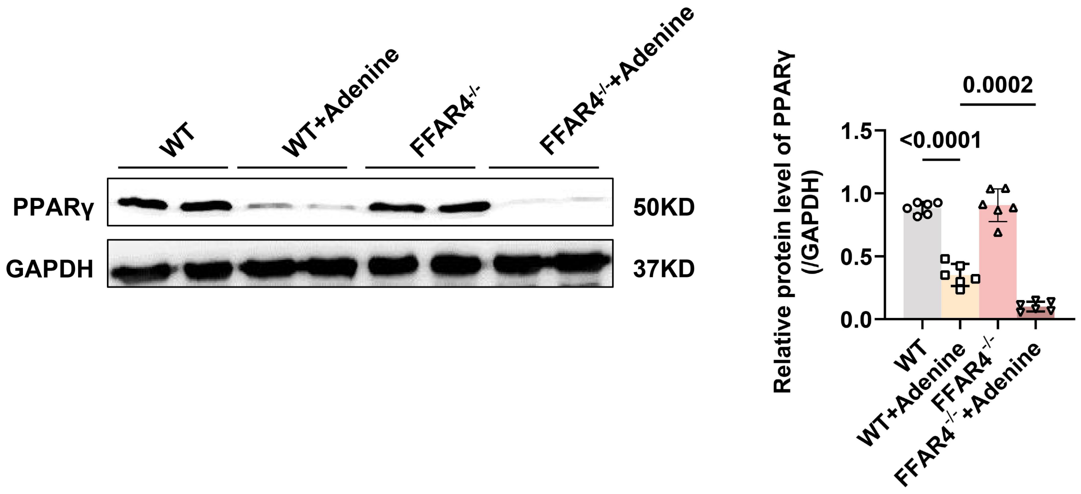

Supplementary Figure 12. FFAR4 regulated expressions of PPARγ in kidneys of adenine diet-induced CKD mice. Protein expressions of PPARγ in kidneys in each group (n=6 per group). Data are presented as mean ± SD.

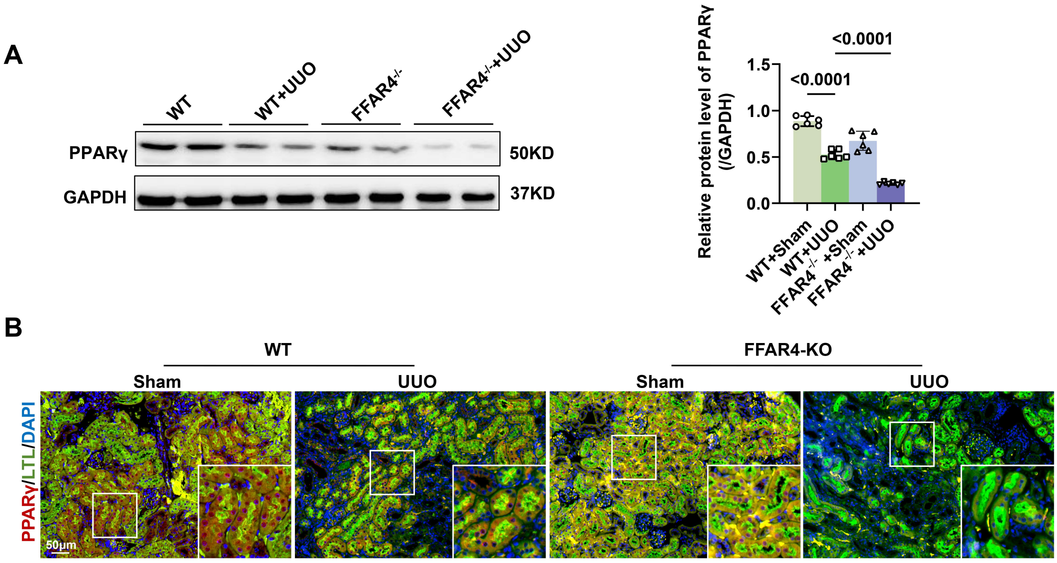

Supplementary Figure 13. FFAR4 regulated expressions of PPARγ in kidneys of UUO mice. A Protein expressions of PPARγ in kidneys in each group (n=6 per group). **B** Representative images of double immunofluorescence for PPARγ and LTL in kidneys of mice. Markers: PPARγ, red; LTL: green; DAPI, blue. Overlay images (PPARγ+ DAPI) appear purple due to the colocation of red and blue markers. Data are presented as mean ± SD.

A

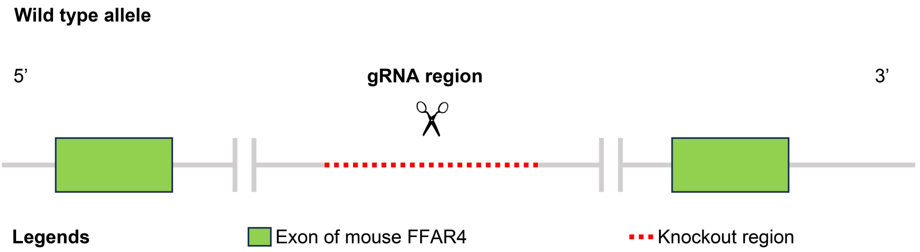

B

Primer Name	Sequence (5’→3’)	PCR size
JS01216-Gpr120-5wt-tF2	AATCCCTCTCCCTAAAGTCACC	-22975bp=223bp
JS01216-Gpr120-3wt-tR2	CCCATGACTGTTTCCTACCCTT	
JS11216-Gpr120-wt-tF2	GGGTCTTCTCAGAGCAGGCTC	Wt=418bp
JS11216-Gpr120-wt-tR2	GTGATGCTCCTGTGTGCCTCA	

C

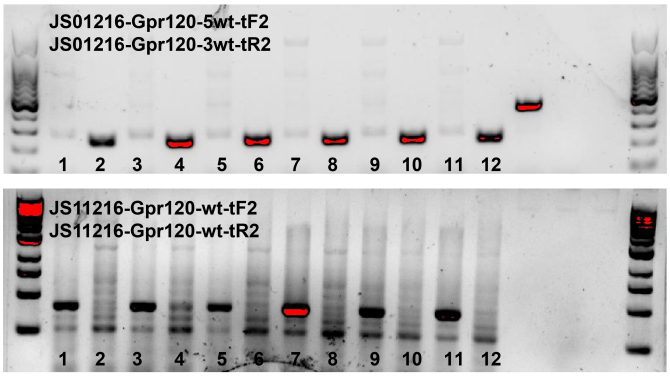

FFAR4 KO/KO: 2, 4, 6, 8, 10, 12
WT/WT: 1, 3, 5, 7, 9, 11

Supplementary Figure 14. Generation of FFAR4-KO mice. A Schematic representation of FFAR4-KO mice generation. **B-C** Identification of the genotype of FFAR4-KO mice by PCR assay.

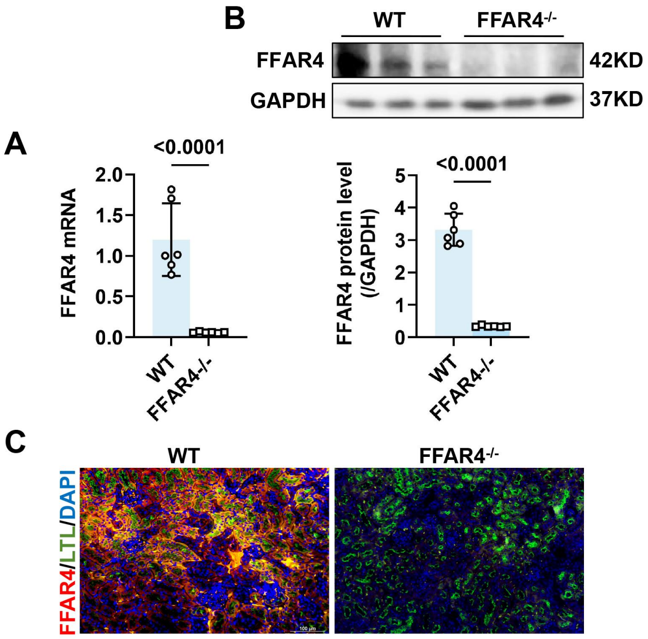

Supplementary Figure 15. Validation of constitutive FFAR4 Knockout. A Relative mRNA levels of FFAR4 of kidneys in each group (n=6 per group). **B** Western Blot analysis of FFAR4 protein expressions of kidneys in each group (n=6 per group). **C** Representative images of double immunofluorescence staining for FFAR4 and LTL in kidneys of WT mice and FFAR4$^{-/-}$ mice. Markers: FFAR4, red; LTL: green; DAPI, blue. Overlay images (FFAR4+LTL) appear yellow due to the colocation of red and green markers.

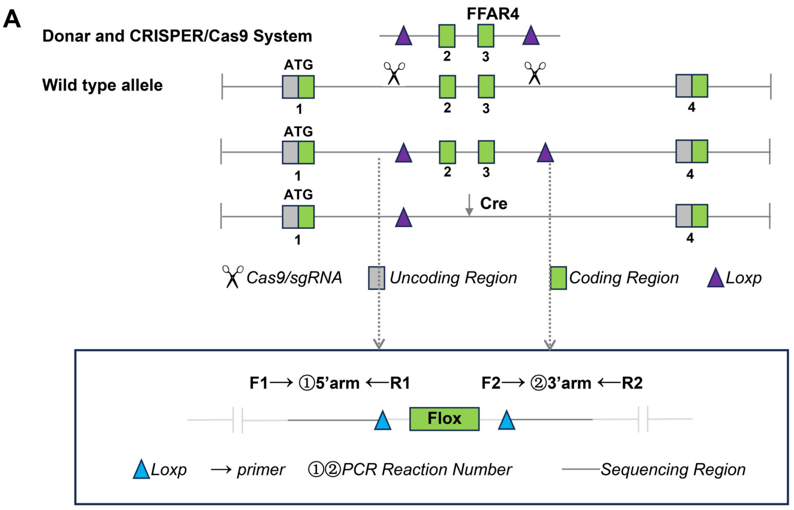

B

Primer Name	Sequence (5'→3')	PCR size	Primer illustration
GJS022020062533-01-Ffar4-5wt-tF1	GTGCAGTTGCCTGTACCTGTAACC	WT:320bp<br>Fl:418bp	FFAR4
GJS022020062533-01-Ffar4-5wt-tR1	GCAGAGCTACTGAAAGGAAAGGC		
T004820-F1	GGGCAGTCTGGTACTTCCAAGCT	KI:353bp	Cdh16-Cre
T004820-R1	ACTGAGTGCCTACTAACCAGCACC		

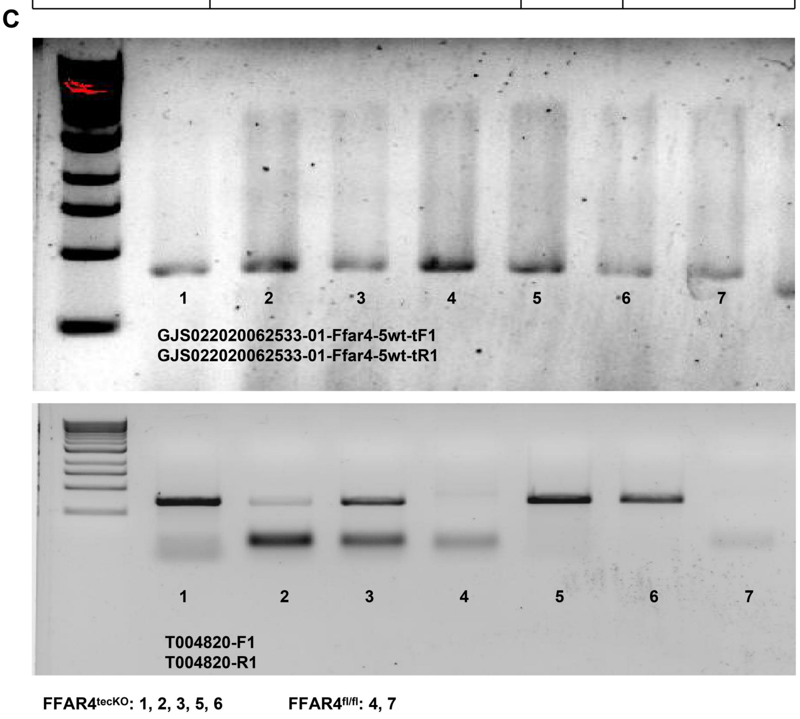

Supplementary Figure 16. Generation of renal tubular epithelial cell-specific (TEC-specific) FFAR4 KO mice. A Schematic representation of FFAR4$^{flox/flox}$ (FFAR4$^{fl/fl}$) mice generation by CRISPR/Cas9-stimulated homologous recombination and design strategy of TEC-specific FFAR4 KO (FFAR4^{tecKO}) mice. **B-C** Identification of the genotype of FFAR4$^{fl/fl}$ mice and FFAR4^{tecKO} (Cdh16-Cre+ FFAR4$^{fl/fl}$) mice by PCR assay.

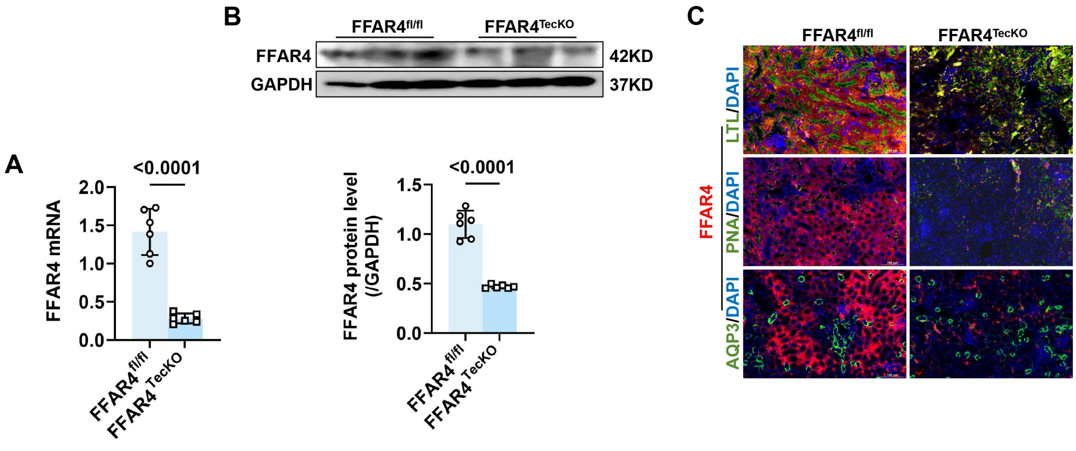

Supplementary Figure 17. Validation of TEC-specific FFAR4 knockout A Relative mRNA levels of FFAR4 of kidneys in each group (n=6 per group). B Western Blot analysis of FFAR4 protein expressions of kidneys in each group (n=6 per group). **C** Representative images of double immunofluorescence staining for FFAR4, LTL, PNA and AQP3 in kidneys of $FFAR4^{fl/fl}$ and $FFAR4^{TecKO}$ mice. Markers: FFAR4, red; LTL, PNA and AQP3: green; DAPI, blue. Overlay images (FFAR4+LTL/ FFAR4+PNA/ FFAR4+AQP3) appear yellow due to the colocation of red and green markers.

Supplementary Table 1. Clinical characteristics of enrolled old and young subjects

Number	Age (year)	eGFR (mL/min/ 1.73m2)	Scr (μmol/L)	group
1	26	101.2	90	Young
2	34	85.25	99	
3	35	79.76	104	
4	35	88.62	97	
5	38	89.4	93	
6	41	73.06	108	
7	41	64.97	119	
1	61	58.84	115	Old
2	63	54.02	122	
3	69	58.06	111	
4	71	40.81	146	
5	71	54.85	115	
6	76	34.99	162	
7	78	59.66	103	
8	81	38.31	146	

eGFR: etimated glomerular filtration rate; Scr, serum creatinine.

Supplementary Table 2. Clinical characteristics of enrolled CKD patients and control subjects

Number	eGFR (mL/min/ 1.73m2)	Scr (μmol/L)	group
1	93.58	88	Control
2	104	56	
3	113.44	43	
4	100.57	66	
5	93.1	48	
1	84.67	108	IgAN
2	94.75	79	
3	33.52	201	
4	83.98	102	
5	81.52	92	
6	60.87	96	
7	51.02	142	
8	72.1	90	
9	55.72	116	
10	85.12	78	
11	69.89	114	
12	42.89	121	
13	69.28	121	
1	31.32	232	HN
2	44.17	160	
3	32.78	217	
4	85.1	98	
5	49.98	113	
6	33.19	189	
7	57.58	127	
8	50.98	131	

9	67.07	95	
10	19.59	338	
11	36.18	200	
12	34.43	217	
13	46.7	161	**HN**
14	29.88	244	
15	56	124	
16	37.36	187	
17	12.95	368	
1	92.79	60	
2	96.44	72	
3	18.7	390	**LN**
4	43.21	144	
5	81.71	71	
1	81.3	85	
2	21.6	276	
3	58.07	119	
4	27.7	234	
5	86.76	91	
6	33.36	196	**DN**
7	71.99	94	
8	43.35	156	
9	70.9	112	
10	13.92	433	
11	28.71	161	

eGFR: etimated glomerular filtration rate; Scr, serum creatinine; IgAN: IgA nephropathy. HN: hypertension nephropathy; LN: lupus nephropathy. DN: diabetic nephropathy.

Supplementary Table 3. The list of primary antibodies.

Name	Company	Catalog Number
Anti-P53 [C8-A11]	HuaAn Biotechnology, Hangzhou, China	M1312-2
Anti-CDKN1A/P21	Affinity Biosciences, Changzhou, China	AF6290
Anti-CDKN2A/p16INK4a	Affinity Biosciences, Changzhou, China	AF5484
Anti-Phospho-Histone H2A.X (Ser139)	Cell Signaling Technology, MA, USA	2577
Anti-fibronectin	HuaAn Biotechnology, Hangzhou, China	ET1702-25
Anti-α-SMA	HuaAn Biotechnology, Hangzhou, China	ET1607-53
Anti-Collagen VI	Abcam, MA, USA	Ab182744
Anti-Collagen I	HuaAn Biotechnology, Hangzhou, China	HA722517
Anti-TGF-β1	HuaAn Biotechnology, Hangzhou, China	HA721143
Anti-Klotho	HuaAn Biotechnology, Hangzhou, China	ET1705-88
Anti-PPARγ	Proteintech Group, Wuhan, China	16643-1-AP
Anti-FFAR4	Abcam, MA, USA	ab223512
Anti-FFAR4	Santa Cruz Biotechnology, CA, USA	sc-390752
Anti-FFAR4	Thermo Fisher Scientific, MA, USA	PA5-50973
Anti-FFAR4	Novus, CO, USA	NBP1-00858
Anti-GAPDH	HuaAn Biotechnology, Hangzhou, China	ET1601-4

Supplementary Table 4. The list of primer sequences.

Mouse Gene	Sequence
Mouse-F-*Cdkn1a*	CCTGGTGATGTCCGACCTG
Mouse-R-*Cdkn1a*	CCATGAGCGCATCGCAATC
Mouse-F-*Cdkn2a*	CGCAGGTTCTTGGTCACTGT
Mouse-R-*Cdkn2a*	TGTTCACGAAAGCCAGAGCG
Mouse-F-*Trp53*	CTCTCCCCCGCAAAAGAAAAA
Mouse-R-*Trp53*	CGGAACATCTCGAAGCGTTTA
Mouse-F-*Fn1*	ATGTGGACCCCTCCTGATAGT
Mouse-R-*Fn1*	GCCCAGTGATTTCAGCAAAGG
Mouse-F-*Col1a1*	TGCCGCGACCTCAAGATGTG
Mouse-R-*Col1a1*	CACAAGGGTGCTGTAGGTGA
Mouse-F-*Col6a1*	CTGCTGCTACAAGCCTGCT
Mouse-R-*Col6a1*	CCCCATAAGGTTTCAGCCTCA
Mouse-F-*Acta2*	CCCAGACATCAGGGAGTAATGG
Mouse-R-*Acta2*	TCTATCGGATACTTCAGCGTCA
Mouse-F-*Lcn2*	GCAGGTGGTACGTTGTGGG
Mouse-R-*Lcn2*	CTCTTGTAGCTCATAGATGGTGC
Mouse-F-*Havcr1*	ACATATCGTGGAATCACAACGAC
Mouse-R-*Havcr1*	ACTGCTCTTCTGATAGGTGACA
Mouse-F-*Il6*	ACAACCACGGCCTTCCCTACTT
Mouse-R-*Il6*	CACGATTTCCCAGAGAACATGTG
Mouse-F-*Tgfb1*	CTCCCGTGGCTTCTAGTGC
Mouse-R-*Tgfb1*	GCCTTAGTTTGGACAGGATCTG
Mouse-F-*Ffar4*	ACCAAGTCAATCGCACCCAC
Mouse-R-*Ffar4*	GTGAGACGACAAAGATGAGCC
Mouse-F-*Gapdh*	GTATGACTCCACTCACGGCAAA
Mouse-R-*Gapdh*	GGTCTCGCTCCTGGAAGATG
